# The AFLATOX^®^ Project: Approaching the Development of New Generation, Natural-Based Compounds for the Containment of the Mycotoxigenic Phytopathogen *Aspergillus flavus* and Aflatoxin Contamination

**DOI:** 10.3390/ijms22094520

**Published:** 2021-04-26

**Authors:** Serena Montalbano, Francesca Degola, Jennifer Bartoli, Franco Bisceglie, Annamaria Buschini, Mauro Carcelli, Donatella Feretti, Serena Galati, Laura Marchi, Nicolò Orsoni, Giorgio Pelosi, Marianna Pioli, Francesco M. Restivo, Dominga Rogolino, Mirco Scaccaglia, Olga Serra, Giorgio Spadola, Gaia C. V. Viola, Ilaria Zerbini, Claudia Zani

**Affiliations:** 1 Department of Chemistry, Life Sciences and Environmental Sustainability, University of Parma, Parco Area delle Scienze 11/A, 43124 Parma, PR, Italy; serena.montalbano@unipr.it (S.M.); jennifer.bartoli@unipr.it (J.B.); franco.bisceglie@unipr.it (F.B.); annamaria.buschini@unipr.it (A.B.); mauro.carcelli@unipr.it (M.C.); nicolo.orsoni@studenti.unipr.it (N.O.); giorgio.pelosi@unipr.it (G.P.); pioli.marianna@gmail.com (M.P.); restivo@unipr.it (F.M.R.); dominga.rogolino@unipr.it (D.R.); mirco.scaccaglia@unipr.it (M.S.); giorgio.spadola1@studenti.unipr.it (G.S.); 2 Interdepartmental Centre for Molecular and Translational Oncology COMT, University of Parma, 43124 Parma, PR, Italy; serena.galati@unipr.it; 3 Department of Medical and Surgical Specialties, Radiological Sciences and Public Health, University of Brescia, 11 Viale Europa, 25123 Brescia, BS, Italy; donatella.feretti@unibs.it (D.F.); gaia.viola@unibs.it (G.C.V.V.); ilaria.zerbini@unibs.it (I.Z.); claudia.zani@unibs.it (C.Z.); 4 Department of Medicine and Surgery, Respiratory Disease and Lung Function Unit, University of Parma, Via Gramsci 14, 43125 Parma, PR, Italy; laura.marchi@unipr.it; 5 Medical Oncology and Breast Unit, Department of Medicine and Surgery, University of Parma, Via Gramsci 14, 43125 Parma, PR, Italy; olga.serra@unipr.it

**Keywords:** aflatoxins, *Aspergillus flavus*, antiaflatoxigenic molecules, antifungals, antimycotoxigenic molecules, new generation fungicides, thiosemicarbazones, crop protection agents

## Abstract

The control of the fungal contamination on crops is considered a priority by the sanitary authorities of an increasing number of countries, and this is also due to the fact that the geographic areas interested in mycotoxin outbreaks are widening. Among the different pre- and post-harvest strategies that may be applied to prevent fungal and/or aflatoxin contamination, fungicides still play a prominent role; however, despite of countless efforts, to date the problem of food and feed contamination remains unsolved, since the essential factors that affect aflatoxins production are various and hardly to handle as a whole. In this scenario, the exploitation of bioactive natural sources to obtain new agents presenting novel mechanisms of action may represent a successful strategy to minimize, at the same time, aflatoxin contamination and the use of toxic pesticides. The Aflatox^®^ Project was aimed at the development of new-generation inhibitors of aflatoxigenic *Aspergillus* spp. proliferation and toxin production, through the modification of naturally occurring molecules: a panel of 177 compounds, belonging to the thiosemicarbazones class, have been synthesized and screened for their antifungal and anti-aflatoxigenic potential. The most effective compounds, selected as the best candidates as aflatoxin containment agents, were also evaluated in terms of cytotoxicity, genotoxicity and epi-genotoxicity to exclude potential harmful effect on the human health, the plants on which fungi grow and the whole ecosystem.

## 1. Introduction

Nowadays, food safety represents a public health priority and deals with the food safeguarding and protection from harmful chemical and biological agents affecting consumer health [1,2]. The contamination of food and feed commodities by harmful fungi has become a recurring human health and security issue [3], since mycotoxigenic species can contaminate cereals and derived products and produce mycotoxins, toxic secondary metabolites typically synthesized by filamentous fungal genera *Aspergillus, Fusarium* and *Penicillium* [4,5]. Among all mycotoxins, aflatoxins (AFs) deserve a particularly high attention because of their acute and chronic hepatotoxicity and their severe carcinogenicity, as reported by the International Agency for Research on Cancer (IARC), that classifies aflatoxins as carcinogenic to humans (Group 1) [6]. Mainly produced by *Aspergillus flavus* and *Aspergillus parasiticus* strains which naturally contaminate the maize fields of several geographic areas, AFs were demonstrated to be influenced, in their occurrence, by a number of critical factors. Several parameters affect *Aspergillus* spp. growth and proliferation on crops: agricultural practices and storage conditions [7,8], water availability [9,10], a range of variables affecting plant health (including the surrounding environment and the extent to which they are protected from pests and diseases) and climatic conditions favorable for fungal spread [11,12]. Aflatoxin contamination risk was generally considered higher in regions characterized by a tropical or subtropical climate, but the impact of climate changes, and in particular the global warming, is currently affecting the distribution of fungal species and therefore the aflatoxin contamination, also in the maize fields of the Po Valley, in Northern Italy [13,14]. Additionally, when considering the health risk represented by mycotoxigenic fungi infecting agricultural commodities, it should be kept in mind that AF contamination is not only limited to raw materials, such as kernels and flours, soybeans, tree nuts, groundnuts, coffee, cocoa and spices [15]: the intake of contaminated feed by livestock could lead to a carrying-over contamination of meat, milk, eggs and derivatives, that could, in turn, be indirect sources of toxin exposure representing a significant threat to human and animal health [16,17,18]. Contamination might be prevented or controlled through various pre- and post-harvest strategies aimed at the containment of AFs that, due to their high stability to most industrial processes, can arrive almost unaltered on the consumer’s table. Different chemical-based approaches have been developed to inhibit conidia germination and fungal growth, or to convert aflatoxins in nontoxic compounds, reducing the post-harvest contamination incidence in food [19]. The use of fungicides is still the most effective and common way to intervene, but this generates well-known concerns about long-term residues in food and in the environment [20]. Natural antifungal compounds such as organic acids, aromatic hydrocarbons, benzimidazoles [21], sterols and aldehydes [22] have been demonstrated to represent a good alternative to synthetic fungicides. For this reason, the design and development of new drugs specifically aimed at preventing the production of aflatoxin with little impact on the environment is to date a topic of primary importance, as recently addressed by various researchers [23,24]. In this context, Alflatox^®^ Project was created, being characterized by a multidisciplinary approach aiming to the identification of new natural scaffold-based antifungal compounds acting directly on the fungal cells and/or on the aflatoxin production, harmless to the environment and to human health [25]. The project was intended to synthetize new compounds starting from natural scaffolds, investigating their possible antifungal and/or anti-aflatoxigenic properties; hence, we merged the biological activities of natural aldehydes or ketones with the well documented protectant capacity of metal ions (Cu^2+^ and Zn^2+^, in particular) against phytopathogens [26]. Condensation with thiosemicarbazide, that easily reacts with the carbonyl group of the natural aldehyde/ketone scaffold and possesses donor atoms suitable to chelate metal ions, was chosen as leading strategy to obtain potentially active compounds. The obtained thiosemicarbazones were then structurally modified to enhance the biological properties (and, in particular, antiaflatoxigenic and antifungal potential) of the parent compounds [23,27,28,29]. Here we report the comprehensive results of the Aflatox^®^ Project, and discuss the properties of the best candidate that we have found and that might be proposed as new generation aflatoxin’s containment agent, safe for the human health and the environment.

## 2. Results

### 2.1. Individuation of Natural Scaffolds and Structural Modifications

**Scheme 1:**
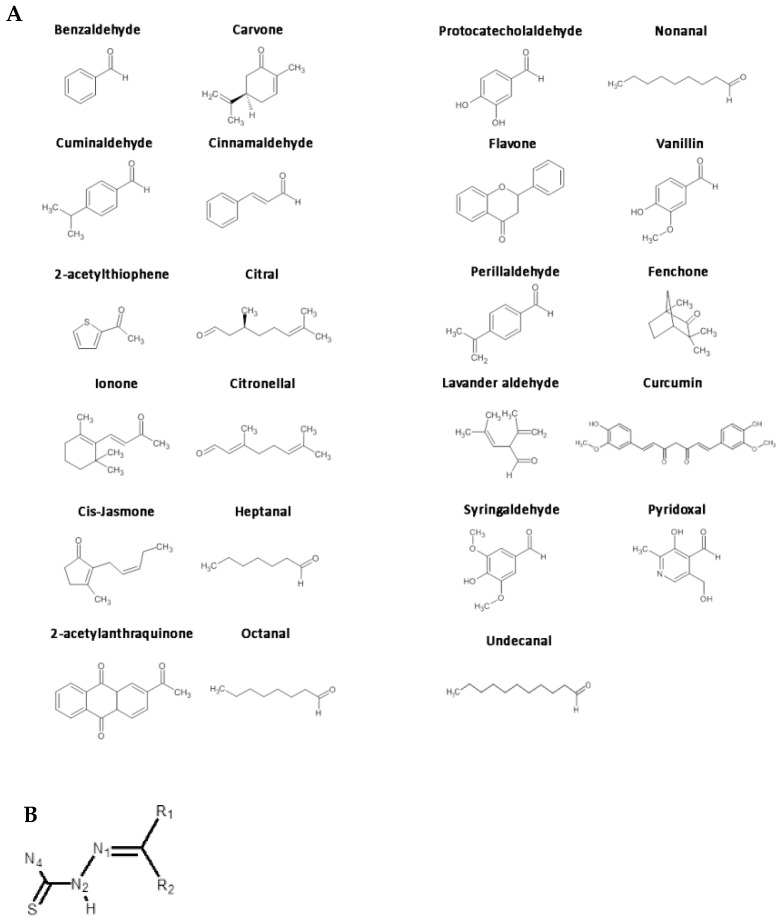
Representation of the structures of parent compounds used to obtain the relevant thiosemicarbazone derivatives investigated in this study (**A**) and the general structure of thiosemicarbazones (**B**).

The Aflatox^®^ Project aimed to identify highly effective compounds against *A. flavus* proliferation and/or aflatoxin biosynthesis [25]. A wide selection of natural aldehydes and ketones (*parent compounds* hereafter; Scheme 1) were condensed with thiosemicarbazide to obtain the thiosemicarbazones (ligands hereafter; TSs). Some starting materials (benzaldehyde, lavandulol and vanillin) were chosen on the basis of their documented antimicrobial activity, since derivatives are widely used as environmentally safe antimicrobial compounds, due to a wide spectrum of inhibitory effects on bacteria, fungi and algae [30,31].

A total of 177 compounds were included in the study, divided in 22 parent compounds, 80 modifications and 75 metal complexes. The molecules were chosen to explore a variety of physicochemical properties such as hydrophobicity, polarity and bulkiness, in order to cover an experimental matrix as large as possible. Compounds were then grouped to create families of derivatives, comprising different TSs obtained taking advantage of the aldehyde/ketone groups, changing the functional groups in N^2^ and N^4^, replacing sulfur with oxygen and by inserting a metal ion to obtain complexes.

### 2.2. Overview of Aflatox^®^ Project Results

#### 2.2.1. Antifungal and Anti-Aflatoxigenic Activity

**Figure 1:**
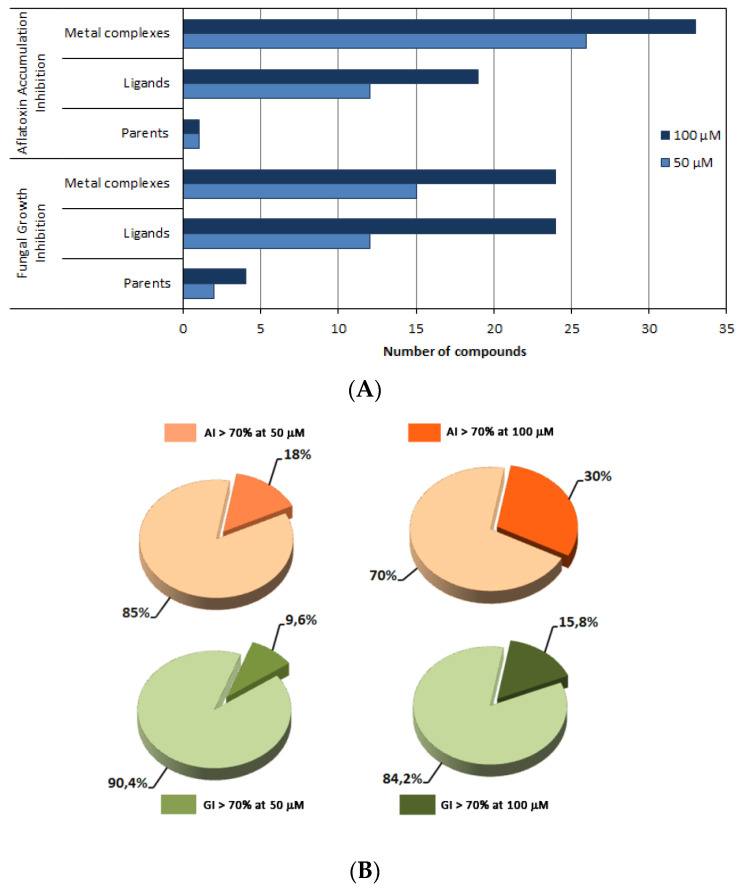
Distribution of active compounds against *A. flavus*. Molecules, grouped by structure (parent compounds, derivatives and metal complexes), were sorted on the basis of their efficacy, at 50 and 100 µM, to inhibit both aflatoxin production (AI) and fungal growth (GI) more than 50% (**A**), and then, without structural distinction, sorted for the ability to inhibit more than 70% (**B**).

The biological effect of compounds was at first evaluated on *A. flavus* in terms of growth inhibition and aflatoxin accumulation containment. Due to their scarce solubility in the coconut-derived medium or interferences with the fluorescence-based detection method (shielding/emission), only 140 of 177 compounds were assayed for aflatoxin inhibition. A total of 28 compounds proved to be effective in inhibiting over 50% the fungal growth at 50 µM concentration, increasing to 51 when tested at 100 µM (Table S1). On the other hand, 39 compounds exerted more than 50% of inhibition on aflatoxin accumulation at 50 µM, and 53% at 100 µM (Figure 1A; Table S2), suggesting, for a few molecules, an interesting anti-aflatoxigenic potential apparently uncoupled from a significant effect on mycelium development. In particular, at 50 µM, 8 compounds resulted in a toxin inhibition higher than 50% while showing a growth inhibition effect lower than 20%. Amid the molecules able to affect the mycelium biomass production, only the 9.6% resulted in effective lowering the fungal growth more than the 70% at 50 µM, versus the 15.8% at 100 µM; the rate of anti-mycotoxigenic compounds exerting more than 70% of inhibition on aflatoxin accumulation increased from 18% to 30%, respectively (Figure 1B).

A great variability of efficacy was observed amongst compounds: derivatives from benzaldehyde, benzophenone, cuminaldehyde, cinnamaldehyde, 2-acetylthiophene and *cis*-jasmone parent compounds proved to be the most active compounds (Tables S1 and S2) [23,27,29,32,33,34], while many others did not show any interesting activity.

#### 2.2.2. Cytotoxic and Genotoxic Activities

The most effective antifungal or anti-aflatoxigenic compounds were submitted to in vitro assays on different cell models (plant, bacterial and human cell lines) to assess their cytotoxic and genotoxic potential, and, therefore, their impact on the environment and human health. A screening of the direct cytotoxicity was performed on human cell lines: for each compound, the dose-response curve was obtained after the treatment of cells for 24 h. The 50% reduction of cell growth (GI_50_) was used to evaluate the cytotoxic potential. A total of 59 compounds was tested: 4 compounds showed a very high cytotoxicity (GI_50_ < 10.0 µM) on at least one cell line, 53 compounds a medium cytotoxicity (10.0 µM < GI_50_ < 50.0 µM; 15 compounds on human colon cell (CRL1790), 21 on human foreskin fibroblast cells (Hs27) and 17 on human fetal lung fibroblast cells (HFL1) cells) and 25 compounds a low cytotoxicity (GI_50_ value > 50.0 µM) on all the tested cell lines. Compounds with the lowest antiproliferative effect on cells were selected to be evaluated for their genotoxic effects, while cytotoxic molecules were excluded from further analyses. After the evaluation of the biological activity with toxicological assays on human cell lines and human leukocytes, 15 compounds were tested for genotoxic and mutagenic activities using tests on human cells, bacteria and plants. Alkaline Comet Assay on U937 cells was performed in order to identify the genotoxic load of molecules that showed any toxic activity: amongst the tested molecules, only 8 did not induce detectable damage to the nuclear DNA. Three compounds (anthraquinone-2-carbonyl-1-thiosemicarbazide, cinnamaldehyde 4,4-dimethylthiosemicarbazone and heptanal thiosemicarbazone) showed a slight mutagenic activity in bacteria; among them, cinnamaldehyde 4,4-dimethylthiosemicarbazone and heptanal thiosemicarbazone also induced a reduction of *A. cepa* root length, together with the anthraquinone-2-carboxylic acid. A slight but significant interference with cell division was recorded for three compounds, and, in particular, for the benzaldehyde 4,4′-dimethylthiosemicarbazone [27], that, without showing any mutagenic effect, induced a lower mitotic index respect to the control. Seven compounds induced DNA damage as chromosomal aberrations in *Allium* root cells at a 10.0 µM concentration: this is, in particular, the case of valerophenone thiosemicarbazone [29], anthraquinone-2-carboxylic acid, cinnamaldehyde 2-methylthiosemicarbazone and 2-formylanthraquinone. The study revealed that only two compounds, that proved to be effective as antifungal and anti-mycotoxigenic agents, resulted not genotoxic, and were thus considered feasible as crop protective agents as discussed below.

#### 2.2.3. Database Creation

All experimental data were collected and used to build a database and, using *InstantJChem* software released by ChemAxon, to create a comprehensive database correlating chemical structures and biological/toxicological activities. Each compound was inserted as a line of an interactive table in which every column matched with an experimental (or calculated) piece of information. Columns were also assembled in sections where data and biological assays were linked together. Database sections were configured as follows:

Chemical structure tool: Allows to view and edit molecular structures. It is particularly useful in the data analysis. It is possible to set up queries which use structures or fragments as input files combined with the chemical or biological properties.

Chemical and physical information: Allows to analyze and find connections between chemico-physical properties of different compounds. Here some interesting parameters such as the molecular weight of the compound, the number of H-bond donor or acceptors and calculated value of logP (partition coefficient) and logS (aqueous solubility) and total polar surface area (TPSA) are reported.

Biological activity on *A. flavus*: Displays the results obtained for fungal growth and aflatoxin biosynthesis inhibition. Data are expressed as percentage of inhibition at different concentrations of compound (50 and 100 μM).

Cytotoxicity: Contains information about the cytotoxic effect of tested compounds, expressed as GI_50_ (50% of cell growth inhibition) values obtained on three different normal cell lines (Hs27, CRL1790 and HFL1 s). In addition, the section reports data obtained on leukemia cell line (U937), that is a cell model to assess drug cytotoxicity already applied in our previous studies [27,28,29,30,31,35].

Genotoxicity: Shows the results of Alkaline Comet Assay, Ames test and *Allium cepa* tests. For Alkaline Comet Assay, the genotoxic effect of the compounds is expressed as DNA percentage in the tail region of the comet (tail intensity, TI), that provides representative data of multiform DNA damage such as single- and double-strand breaks, DNA cross-links, base damage and alkali-labile sites [36]. The Ames test assesses the compound’s ability to induce mutations in *Salmonella typhimurium* DNA, and data are expressed as mutagenicity ratio (MR) dividing the revertants/plate by spontaneous mutation rate [37,38,39]. On *A. cepa* both toxicity and genotoxicity were detected. Toxicity results are expressed as EC_50_, i.e., the concentration at which the compound induces the 50% of *A. cepa* root growth inhibition. The *A. cepa* genotoxicity test detects chromosomal damage as micronuclei (MN) and chromosomal aberrations (CA) [40,41,42]. The Database files is available at: https://aflatox.unibs.it (accessed on 26 April 2021).

### 2.3. Exploring the 2-Acetylthiophene Group

A cross-check of the outcome from biological analyses led to the individuation of the 2-acetylthiophene thiosemicarbazone (2-actpTS) as the best candidate for representing an effective agent of control for *A. flavus* without toxicity potential toward the human health, since it proved to efficiently inhibit aflatoxin production while not showing negative effect in terms of cytotoxicity and genotoxicity. Two diverse strategies have then been tried starting from 2-actpTS: the sulfur atom in the heterocycle ring was substituted obtaining 2-acetylfuran (2-acfurTS), and the copper(II) and zinc(II) complexes were synthesized.

#### 2.3.1. Antifungal and Anti-Aflatoxigenic Activity

**Scheme 2:**
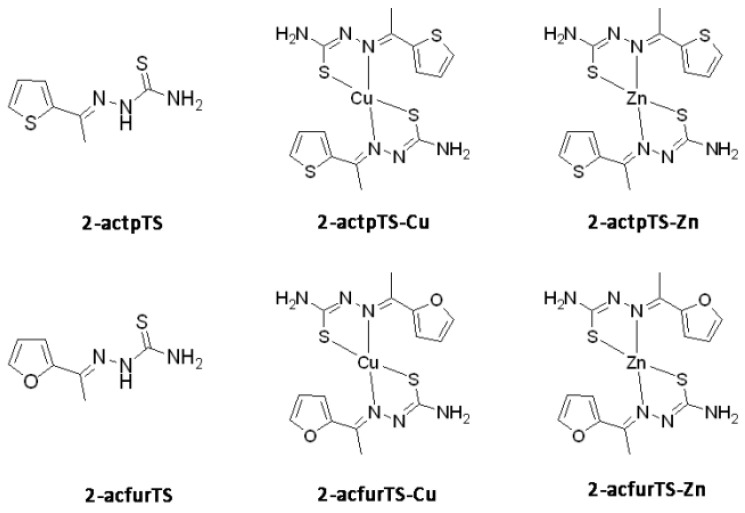
Representation of the structures of 2-acetylthiophene thiosemicarbazone (2-actpTS), 2-acetylfuran thiosemicarbazone (2-acfurTS) and their Cu(II) and Zn(II) complexes.

The two ligands 2-actpTS and 2-acfurTS were complexed with Cu(II) and Zn(II) ions, and compared for their biological activities (Scheme 2).

**Figure 2:**
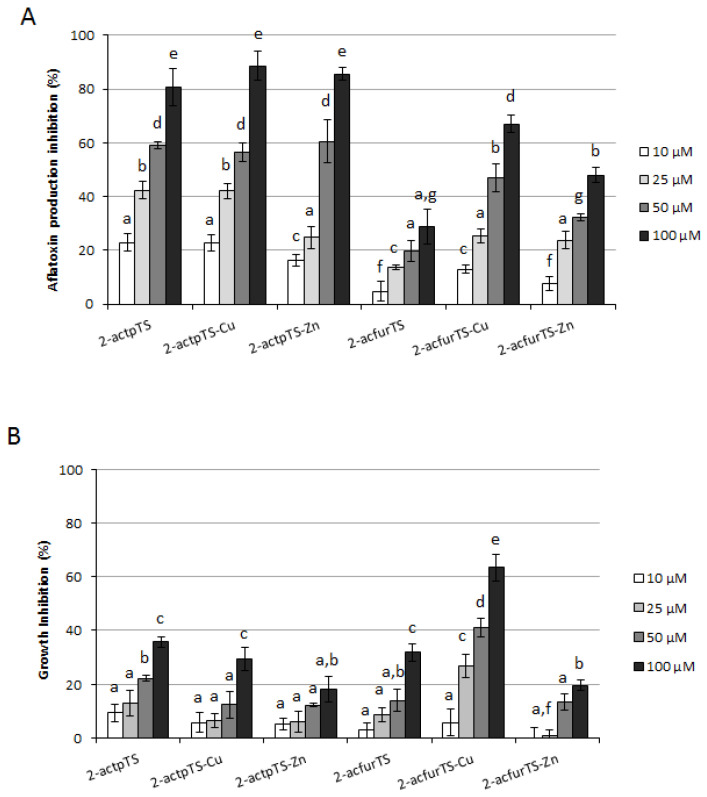
Effects of 2-actpTS, 2-acfurTS and their Cu(II) and Zn(II) complexes on the aflatoxin accumulation (**A**) and fungal growth (**B**) of *A. flavus*. Culture medium was amended with an increasing concentration of molecules (from 10 to 100 μM); values are reported as inhibition percentage as compared to the control (DMSO amended cultures) ± S.D. Different letters indicate statistically significant differences (*p* < 0.05).

When the antifungal and anti-aflatoxigenic potential of 2-actpTS and 2-acfurTS were evaluated, a dose-dependent effect was found. However, despite of a similar, modest inhibitory activity on fungal growth, 2-actpTS resulted in a higher anti-toxigenic activity respect to 2-acfurTS (Figure 2): in fact, while 2-actpTS percentage of aflatoxin accumulation inhibition ranged from 23% at the lowest concentration (10 μM) to 80% at the highest (100 μM), *A. flavus* 2-acfurTS treated cultures were inhibited at a maximum of 28.7%. With the aim of enhancing their biological potential, the TSs were used in their complexed form with Cu^2+^ and Zn^2+^ ions. Generally, the containment effect of 2-actpTS-Cu and 2-actpTS-Zn on aflatoxin production did not differ from the parent compound (only for 2-actpTS-Zn at 25 μM a significant activity decrease was observed), while an increase of anti-aflatoxigenic activity of 2-acfurTS was obtained after the complexation (Figure 2A), and Cu^2+^ provided the best performance improvement. With regard to the impact on fungal growth, the metal complexes of 2-actpTS resulted slightly less effective than their parent, as observed also for 2-acfurTS-Zn (at 100 μM); on the contrary, 2-acfurTS-Cu showed a higher fungistatic activity respect to 2-acfurTS, at each concentration (Figure 2B).

#### 2.3.2. Cytotoxic Activity

**Figure 3:**
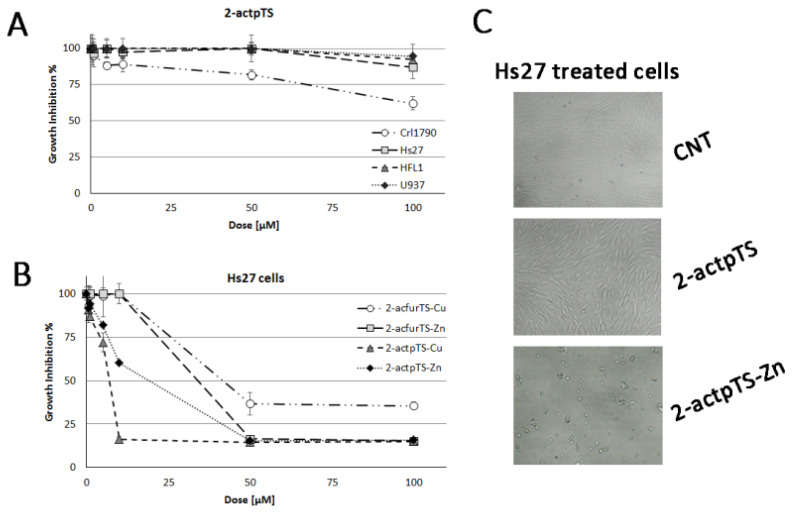
Antiproliferative activity of 2-actpTS on a panel of human healthy cell lines (human colon cell Crl1790, human foreskin fibroblast cells Hs27 and human fetal lung fibroblast cells HFL1) and a leukemia cell line (U937) (**A**). Antiproliferative activity of 2-actpTS-Cu, 2-actpTS-Zn, 2-acfurTS-Zn and 2-acfurTS-Cu on Hs27 cells after 24 h treatment (**B**). Data are expressed as growth inhibition percentage compared to the negative control (DMSO-treated cells; CNT). Example of Hs27 cells after 24 h treatment with 100 µM 2-actpTS and 2-actpTS-Zn obtained with in vitro real time microscopy (JuLI Smart fluorescent cell analyzer instrument, Digital Bio Technology, Boston, USA) (Magnification: 4×, NA: 0.16) is shown (**C**).

The 2-acfurTS compound was excluded from the cytotoxic assay due to its low effect on aflatoxin accumulation. As a first step, all the other compounds were tested on Hs27 cells to evaluate their antiproliferative activity. As reported in Figure 3A (Table S3), 2-actpTS, the most powerful antifungal and anti-aflatoxigenic compound, did not show cytotoxic effect on Hs27 cell line after 24 h treatment, and was hence evaluated on additional healthy and cancer cell lines (Crl1790, HFL1 and U937). After 24 h treatment, 2-actpTS did not induce 50% inhibition of cell proliferation against neither HFL1 nor U937 cells, while a mild cytotoxic activity was detected against Crl1790 cells, even if only at the highest concentration (100 µM). On the contrary, the metal complexes of 2-actpTS, 2-actpTS-Cu and 2-actpTS-Zn induced a strong antiproliferative activity, showing GI_50_ values equal to 6.2 µM and 15.4 µM after 24 h treatment, respectively (Figure 3B). Additionally, 2-acfurTS-Zn and 2-acfurTS-Cu induced an antiproliferative effect on Hs27 cells after 24 h treatment, with GI_50_ value of 30.5 µM and 39.0 µM (Figure 3B; Table S4).

#### 2.3.3. Genotoxic Activity

**Figure 4:**
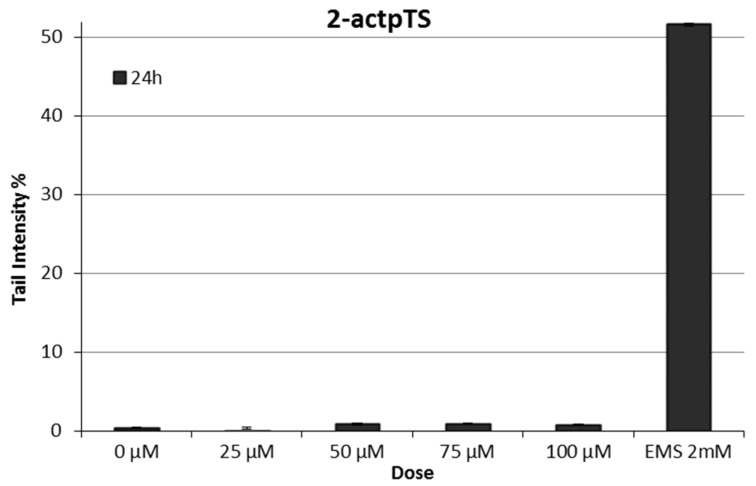
Genotoxicity activity of 2-actpTS detected by Alkaline Comet Assay on U937 cells treated for 24 h. As positive control was used EMS (2 mM). Data are expressed as tail intensity percentage. The *p*-value was fixed at 0.001.

The ability of 2-actpTS to induce DNA damage on human cells was evaluated in the Alkaline Comet Assay. After 24 h treatment, we did not detect single and double DNA strand breaks and alkali-labile sites: the molecule did not induce significant DNA damage (Figure 4).

The results of the Ames test on *S. typhimurium* TA98 and TA100 strains, expressed as revertants/plate and as MR, are reported in Table 1. The compound showed no mutagenicity at any of the concentrations tested on TA98 and TA100 strains, both without (−S9) and with (+S9) metabolic activation. *A. cepa* toxicity test showed that the EC_50_ approximately corresponded to the highest concentration (100 µM). Table 2 shows the frequency of micronuclei and chromosomal aberrations in root cells, in duplicate experiment. The test of micronuclei was performed only at 10, 25 and 50 µM because the 100 µM dose resulted highly toxic for all samples: the roots were strongly damaged and could not be analyzed. No increase in micronuclei and chromosomal aberrations frequency was observed in all the tested doses.

## 3. Discussion

Mycotoxin contamination is considered a global public health problem and represents a challenge to food safety. Aflatoxins, in particular, can accumulate in food during agricultural, storage and processing practices, and, due to their great resistance to conventional treatments usually applied in industrial processing, their persistence in food commodities is prolonged. Thus, several strategies have been proposed to prevent mycotoxins contamination/diffusion at different levels of the food chain. However, despite the wide efforts addressed by the scientific community, no definitive, clear-cut solutions have been found yet [43]. To date, one of the most promising strategies relies on the prevention of mycotoxigenic fungal species diffusion on crops, or, at least, on their inhibition in accumulating mycotoxins on the plant material; therefore, the design of fungicide/fungistatic compounds is more challenging, because we must also consider the requirements of sustainability and environmental-friendly solutions, drug resistance occurrence in microbial pathogens and the problem of residual toxicity in using conventional chemicals. In this scenario, natural-based bioactive compounds are regarded as a valuable “treasure chest” to be searched for the individuation of promising antifungals/anti-mycotoxigenic compounds. In fact, since natural products whose biological activity has been already characterized are mainly secondary metabolites synthesized by organisms to protect themselves from both abiotic and biotic stressors—such as natural antagonists, predators or pathogens –, the exploitation of their scaffolds is regarded as a privileged starting point for the design of highly specific and effective new antifungals. In turn, the pre-existing biological activity could be enhanced by specific structural modifications. Several researches have proposed natural compounds or their chemical analogues as crop-protective agents; additionally, metallic ions such as copper and zinc have been long time used for their fungicidal properties, demonstrating containment effects on the growth of phytopathogenic fungi and mycotoxins production [27,29,34]. In our study, we screened an extended panel of natural compounds and synthesized derivatives in order to individuate—and possibly ameliorate—effective antifungal and/or anti-aflatoxigenic molecules against *A. flavus*, being reasonably safe for the health of the stakeholders and the environment. Molecules with antimicrobial activity often share some structural characteristics: for example, low molecular weight, aromatic rings, or the presence of sulfur atoms are found in a number of known antifungals. Thiosemicarbazones are extremely versatile sulfur-containing molecules whose peculiar and extensive electronic delocalization, as well as the presence of a thione-thiol tautomerism, have been indicated as the cause of their biological activity [44], perfectly fit with these assumptions. The introduction of thio- group has long time been known to determine a general increase of biological effects, frequently demonstrating that ligands exhibit more interesting characteristics than the relevant parent compounds (Figure 1A). In this sense, we found interesting the differences observed in the biological activity of 2-acetyl-thiophene and 2-acetyl-furan rings. The furan ring is found in a large number of biologically active, natural molecules, and many furan-containing derivatives exhibit extensive pharmacological activities [45]; indeed, 2-acetyl-furan has been detected as a volatile component of pine leaves, French lavender and chestnuts honey, that proved to possess high antimicrobial potential [46], and has been demonstrated to be also the most abundant compound characterizing the *Perilla frutescens* essential oils that, tested against different *Aspergillus* species comprising *A. flavus*, showed potent antifungal effect [47]. However, our data showed that, while 2-acetyl-thiophene and 2-acetyl-furan are fundamentally inactive, their thiosemicarbazones inhibit aflatoxin biosynthesis in *A. flavus*. In particular, 2-acetyl-thiophene thiosemicarbazone was more active than 2-Acetyl-furan thiosemicarbazone, suggesting that the sulfur atom on the ring provides an additional anti-toxigenic effect.

Our interest in TSs also relied on the ease with which their structure can be modified, and their suitability to act as bidentate ligands towards metal ions, using the sulfur and the iminic nitrogen and thus forming a five-term coordination ring. TSs chain can be modified with the addition of other fragments at the thiosemicarbazide aminic nitrogen position, to tune the hydrophobicity/hydrophilicity of the whole molecule. In fact, the choice of a polar substituent rather than a lipophilic one, can markedly modulate the molecule hydrophobicity, for example acting on the molecule solubility in aqueous media or influencing the absorption in lipophilic substrates. Moreover, the choice of electron-donor rather than electron-withdrawing groups as substituents, can modulate the coordinative behavior enhancing or reducing the electron delocalization within the thiosemicarbazonic chain [48]. When the N^2^ is not alkylated, TSs can also act as an anionic ligand through the de-protonation of the hydrazinic nitrogen (N^2^). Due to the presence of mixed hard-soft N-S donor atoms, TSs show high affinity to different metals, whereas the N^2^ de-protonation can contribute to balance the positive charges of the metal ion, providing electrostatic properties that make chelation of both transition elements and alkaline or alkaline earth metals possible. The first example of thiosemicarbazonic compounds tested as antifungal agents dates back to 1960 [49], when Benns and collaborators evaluated a panel of 40 TSs and their copper complexes against *Aspergillus niger*: results evidenced that some TSs possessed a higher antifungal potential if compared with the two commercial fungicides used as reference, and that only a few ligands were more effective than their corresponding metal complexes, indicating that the antifungal activity might sometimes be exerted by the ligand structure. Accordingly, we reported that various TSs metal complexes, coordinated with copper, nickel and zinc atoms, synergistically improved the antifungal/anti-aflatoxigenic properties of the relative ligands against *A. flavus*: this is the case, for example, of cuminaldehyde and cinnamaldehyde derivatives, and, as previously described, of benzaldehyde [27] and benzophenone [29]. On the other hand, if this observation was confirmed also by Cu and Zn complexes of 2-acfuTS, that both resulted more active in lowering AFs production than their ligand, it apparently contrasts with what has been obtained with 2-actpTS-Cu and-Zn, as effective as 2-actpTS. However, the hypothesis of the copper ion thought to be the redox active center of thiosemicarbazone compounds—in terms of both antifungal and anti-mycotoxigenic effect—has been already confuted (as in the case of *cis*- and dihydrojasmone thiosemicarbazones [34]), being suggested to mainly rely on the features of parent molecules. Hence, if purposive modifications could be applied in order to raise the molecules selectivity and their activity power, the in vivo validation should be performed each time. Anyway, even if coordination to Cu(II) and Zn(II) could enhance the anti-aflatoxigenic properties of TS alone (see for example 2-acfuTS-Cu and-Zn), the metal ions tend also to confer to the complexes cytotoxic characters that make them practically useless.

Interestingly, amongst all the screened TSs, the highest number of biologically active compounds refers to anti-aflatoxigenic molecules, indicating that probably the majority of molecular/cellular targets belong to secondary metabolism pathways, and thus only in few cases they might severely affect the fungal growth. More remarkably, some molecules were found to show anti-toxigenic potential uncoupled with a significant effect on mycelium development [23,33,34,50], this is considered particularly interesting in an ecological perspective overall. These selective anti-aflatoxigenic compounds could become considerably interesting for applications aimed at excluding ordinary fungicidal, nonspecific agents.

## 4. Materials and Methods

### 4.1. Chemistry and Syntheses

All reactants used were purchased form Sigma Aldrich. The ^1^H-NMR spectra were recorded on a Bruker Anova spectrometer at 400 MHz. ESI-MS analysis were performed using a Waters Acquity Ultraperformance ESI-MS spectrometer with Single Quadrupole Detector. Elemental analyses were performed on a CHNS ThermoFischer (Rodano, MI, Italy).

Thiosemicarbazones are synthesized following literature methods or as reported in the authors’ previous papers [23,25,27,28,29,32,33,34,35]. In general, an equimolar amount of thiosemicarbazide was mixed with the appropriate aldehyde or ketone in absolute ethanol, with a small amount of acetic acid as a catalyst. The mixture was refluxed under stirring. The precipitate was filtered out, washed with cold ethanol and dried under vacuum. The metal complexes were synthesized following this general procedure: the ligand was mixed with the metal salt in ethanol using a metal to ligand ratio of 1:2. The mixture was left under stirring at room temperature for 2 h. Usually a change in the solution color was observed during the reaction. Finally, the solvent was removed under reduced pressure; the product was washed with diethyl-ether, and then dried under vacuum.

The characterizations and synthesis of the newly synthesized compounds 2-actpTS, 2-acfurTS, and their Cu and Zn complexes, that are not reported elsewhere, as follows.

*2-acetylthiophenethiosemicarbazone (2-actpTS):* Thiosemicarbazide (0.36 g, 3.9 mmoL) was dissolved in 20 mL of hot absolute ethanol, with 1 mL of acetic acid as catalyst. 2-acetylthiophene (0.50 g, 3.9 mmoL) was added dropwise to the hot solution. The yellow mixture was reflux overnight under stirring. The day after a precipitate was observed. The mixture was stored at −4 °C for 24 h and then filtered out. The precipitate was washed with cold ethanol and diethyl-ether. Yellow powder. Yield: 94%. ^1^H-NMR (δ, ppm; DMSO-d_6_): 2.33 (s, 3 H), 7.08 (dd, 1 H, ^3^J_1_ = 5.1 Hz, ^3^J_2_ = 3.7 Hz), 7.43 (s, 1 H), 7.52 (dd, 1 H, ^3^J_2_ = 3.7 Hz, ^4^J = 1.2 Hz), 7.58 (dd, 1 H, ^3^J_1_ = 5.1 Hz, ^4^J = 1.2 Hz), 8.30(s, 1 H), 10.33 (s, 1 H). ^13^C-NMR (δ, ppm; DMSO-d_6_): 15.2, 128.2, 128.5, 129.1, 143.3, 145.4, 179.0. CHNS analysis: C_7_H_9_N_3_S_2_: C: 42.19, H: 4.55, N: 21.09. S: 32.17; found: C: 42.24, H: 4.30, N: 21.03, S: 32.41. ESI-MS (+) m/z calc. 200.02, found 200.25. IR: 3356 cm^−1^, 3246 cm^−1^ and 3135 cm^−1^ ν NH, 1655 cm^−1^ ν C = N, 1501 cm^−1^ ν C = C, 1052 cm^−1^ and 881 cm^−1^ ν C = S.

*2-acetylfuranthiosemicarbazone (2-acfurTS)*: Thiosemicarbazide (0.50 g, 4.5 mmoL) was dissolved in 20 mL of hot absolute ethanol, with 1 mL of acetic acid as catalyst, 2-acetylfuran (0.41 g, 4.5 mmoL) was added dropwise to the hot solution. The orange mixture was reflux overnight under stirring. The day after a precipitate was observed. The mixture was stored at −4 °C for 24 h and then filtered out. The precipitate was washed with cold ethanol and diethyl-ether. Orange powder. Yield: 46%. ^1^H-NMR (δ, ppm; DMSO-d_6_): 2.24 (s, 3 H), 6.59 (dd, 1 H, ^3^J_1_ = 3.4 Hz, ^3^J_2_ = 1.8 Hz), 7.11 (dd, 1 H, ^3^J_1_ = 3.4 Hz, ^4^J = 0.8 Hz), 7.71 (s, 1 H), 7.77 (dd, 1 H, ^3^J_2_ = 1.8 Hz, ^4^J = 0.8 Hz), 8.30 (s, 1 H), 10.30 (s, 1 H). ^13^C-NMR (δ, ppm; DMSO-d_6_): 13.8, 111.1, 112.6, 140.6, 144.8, 152.2, 179.2. CHNS analysis: C_7_H_9_N_3_OS: C: 45.89, H: 4.95, N: 22.93. S: 17.50; found: C: 45.80, H: 4.51, N: 23.04, S: 17.90. ESI-MS (+) m/z calc. 184.04, found 183.95. IR: 3415 cm^−1^, 3235 cm^−1^ and 3137 cm^−1^ ν NH, 1631 cm^−1^ ν C = N, 1575 cm^−1^ ν C = C, 1098 cm^−1^ and 828 cm^−1^ ν C = S.

*Bis(2-acetylthiophenethiosemicarbazonato) copper(II) (2-actpTS-Cu)*: 2-actpTS (0.10 g, 0.50 mmoL) was dissolved in 20 mL of methanol and copper(II) acetate (0.05 g, 0.025 mmoL) was dissolved in 2 mL of methanol. The metal was added dropwise to the stirring solution. The solution immediately turned to pale green. The solution was left under stirring for 2 h. The solvent was removed under reduced pressure and the product was washed with diethyl-ether. Green powder. Yield: 59%. ESI-MS (+) m/z calc. 460.59, found 461.09. CHNS analysis: C_14_H_16_CuN_6_S_4_: C: 36.55, H: 3.51, N: 18.27 S: 27.87; found: C: 36.45, H: 3.53, N: 18.47, S: 28.25. IR: 3397 cm^−1^ and 3114 cm^−1^ν NH, 1610 cm^−1^ ν C = N, 1545 cm^−1^ ν C = C, 1079 cm^−1^ and 819 cm^−1^ ν C = S.

*Bis(2-acetylthiophenethiosemicarbazonato) zinc(II) (2-actpTS-Zn*): 2-actpTS (0.10 g, 0.50 mmoL) was dissolved in 20 mL of methanol and zinc(II) acetate (0.06 g, 0.25 mmoL) was dissolved in 2 mL of methanol. The metal was added dropwise to the stirring solution. The solution immediately turned color. The solution was left under stirring for 2 h and a precipitate was observed. The mixture was stored at −4 °C for 24 h and then filtered out. The product was washed with cold methanol and diethyl-ether. Pale yellow powder. Yield: 74%. ^1^H-NMR (δ, ppm; DMSO-d_6_): 2.43 (s, 6 H), 7.09 (s, 4 H), 7.17 (dd, 2 H, ^3^J_1_ = 5.1 Hz, ^3^J_2_ = 3.9 Hz), 7.69 (dd, 2 H, ^3^J_2_ = 3.9 Hz, ^4^J = 1.2 Hz), 7.89 (dd, 2 H, ^3^J_1_ = 5.1 Hz, ^4^J = 1.1 Hz). ^13^C-NMR (δ, ppm; DMSO-d_6_): 22.2, 126.5, 132.2, 135.5, 149.2, 172.26. ESI-MS (+) m/z calc. 462.94, found 461.76. CHNS analysis: C_14_H_16_N_6_S_4_Zn: C: 36.40, H: 3.49, N: 18.19 S: 27.78; found: C: 36.34, H: 3.55, N: 18.02, S: 28.18. IR: 3447 cm^−1^ and 3303 cm^−1^ ν NH, 1593 cm^−1^ ν C = N, 1557 cm^−1^ ν C = C, 834 cm^−1^ ν C = S.

*Bis(2-acetylfuranthiosemicarbazonato) copper(II) (2-acfurTS-Cu)*: 2-acfurTS (0.11 g, 0.57 mmoL) was dissolved in 20 mL of methanol and copper(II) acetate (0.06 g, 0.028 mmoL) was dissolved in 2 mL of methanol. The metal was added dropwise to the stirring solution. The solution immediately turned to dark green. The solution was left under stirring for 2 h. The solvent was removed under reduced pressure and the product was washed with diethyl-ether. Dark green powder. Yield: 75%. ESI-MS (+) m/z calc. 428.98, found 428.11. CHNS analysis: C_14_H_16_CuN_6_O_2_S_2_Cu: C: 39.29, H: 3.77, N: 19.64 S: 14.98; found: C: 39.02, H: 3.64, N: 19.52, S: 15.33. IR: 3421 cm^−1^ and 3297 cm^−1^ν NH, 1575 cm^−1^ ν C = N, 816 cm^−1^ ν C = S.

*Bis(2-acetylfuranthiosemicarbazonato) zinc(II) (2-acfurTS-Zn)*: 2-acfurTS (0.10 g, 0.55 mmoL) was dissolved in 20 mL of methanol and zinc(II) acetate (0.06 g, 0.27 mmoL) was dissolved in 2 mL of methanol. The metal was added dropwise to the stirring solution. The solution immediately turned orange. The solution was left under stirring for 2 h and a precipitate was observed. The mixture was stored at −4 °C for 24 h and then filtered out. The product was washed with cold methanol and diethyl-ether. Pale orange powder. Yield: 82%. ^1^H-NMR (δ, ppm; DMSO-d_6_): 2.41 (s, 6 H), 6.54 (dd, 2 H, ^3^J_1_ = 3.6 Hz, ^3^J_2_ = 1.8 Hz), 6.75 (sb, 4 H), 6.95 (tb, 2 H, ^3^J_1_ = 3.6 Hz), 7.19 (tb, 2 H, ^3^J_2_ = 1.9 Hz). ESI-MS (+) m/z calc. 430.82, found 431.42. ^13^C-NMR (δ, ppm; DMSO-d_6_): 17.0, 113.0, 113.8, 144.0, 146.2, 145.0, 174.3. CHNS analysis: C_14_H_16_N_6_O_2_S_2_Zn: C: 39.12, H: 3.75, N: 19.55 S: 14.92; found: C: 39.24, H: 3.83, N: 19.49, S: 14.75. IR: 3415 cm^−1^ and 3297 cm^−1^ ν NH, 1607 cm^−1^ ν C = N, 1587 cm^−1^ ν C = C, 825 cm^−1^ ν C = S.

### 4.2. Biological Assays

#### 4.2.1. Determination of Fluorescence Emission/Shielding of Compounds

Before being tested for their biological activity, compounds were evaluated for their possible interference with the direct fluorescence-based detection of AFs in the CCM culture medium; emission and absorption at λ_ex_ = 360 nm (aflatoxins specific excitation wavelength) and λ_em_ = 465 nm (aflatoxins specific emission wavelength) were measured directly in the CCM medium used for the toxin accumulation assay, using a fluorescence microplate reader (SPECTRAFluor PLUS, TECAN, Männedorf, Swiss). Compounds were tested at the final concentration of 50 and 100 μM; molecules showing emission/shielding values over 20% were discarded for the following analyses.

#### 4.2.2. Aspergillus Flavus Assays

The *A. flavus* aflatoxigenic strain CR10 and the non-toxigenic strain TOφ were used and are available by Authors under request [27]. Fungal strains were maintained and cultured for sporulation on YES 5% agar medium [2% (*w*/*v*) yeast extract; 5% (*w*/*v*) sucrose; 2% (*w*/*v*) agar] [32].

##### Germination and Early Growth Determination

The first stage of mycelium development was evaluated using a multi-well plate system (Tissue Colture Plate 96-wells Flat Bottom with Lid, Sarstedt, Numbrecht, Germany), as previously reported by Bartoli et al. 2019 [29]: briefly, 5 × 10^3^ spores were inoculated in a final volume of 200 μL of YES 5% liquid medium in each well amended with the molecules test; compounds were tested at increasing concentration, ranging from 10 to 100 μM, and DMSO was used as control. Microplates were incubated at 28 °C in stationary conditions, in the dark, then the optical density was measured at 620 nm after 46–48 h from the inoculum, using a microplate reader (MULTISKAN EX, Thermo Electron Corporation, Finland). Four replicates were performed for each treatment, and experiments were conducted in triplicate. Data were expressed as percentage of growth inhibition with respect to control.

##### Aflatoxin Accumulation Assessment

The high-throughput method for the detection and the quantitation of aflatoxin accumulated in the culture medium described by Degola et al. was used [51]. Spore suspensions of toxigenic or non-toxigenic strains (5 × 10^2^ spore/well) were inoculated in a 96-well microplate, in a final volume of 200 μL/well of coconut clarified medium supplemented with the compounds at increasing concentrations (from 10 to 100 μM). Equivalent cultures added with DMSO were used as control. Plates were incubated in static conditions at 25 °C in the dark for 6 days; the amount of aflatoxin released in the medium was detected by a fluorescent microplate reader (SPECTRAFluor PLUS, TECAN, Männedorf, Swiss). Four replicates were performed for each condition, and experiments were conducted in triplicate. Data were expressed as percentage of aflatoxin inhibition with respect to control.

#### 4.2.3. Cytotoxicity and Genotoxicity Assessment

##### Cytotoxicity Assay

The best antifungal and/or antiaflatoxigenic compounds were tested to determine their cytotoxic activity against human normal cell lines deriving from lung (HFL1, ATCC and CCL-153), skin (Hs27, ATCC, CRL1634) and colon (Crl1790, ATCC, CCD 841 CoN) and a cell line deriving from a hematological tumor (U937, ATCC and CRL-3253). These different cell lines have been chosen to simulate the different human exposition routes to pesticide. Cell culture conditions have been previously reported [29]. Compound cytotoxicity was determined using CellTiter 96^®^ AQueous One Solution Cell Proliferation Assay (Promega Corporation, Madison, WI, USA). The very detailed protocol was described by Bartoli et al. [29]. In brief, cells were seeded in a 96-well plate at a density of 5000 cells/well and after 24 h cells were treated with different concentrations of the compounds (0.5–1–5–10–50–100 µM). After the treatment period (24 h), the CellTiter Reagent was added to each well (20 µL/well) and the plate incubated for 4 h at 37 °C. The plate was then read at 485 nm in a multiwell plate reader (TECAN SpectraFluor Plus, Männedorf, Switzerland). The GI_50_ value, defined as the concentration of the compound required to inhibit cell proliferation by 50% relative to untreated cells, was determined from the dose-response curve for each cell line.

##### Genotoxicity Assay

Our standard protocol for the Alkaline Comet Assay (pH > 13) was previously described in great detail [27,28,29,30,31,32]. The DNA-damaging effects of non-cytotoxic molecules were evaluated on U937 cells using 25 μM as the lowest concentration and 100 µM as the highest for 24 h treatment. After exposure, cell viability was checked using the trypan blue exclusion method: a viability of at least 70% compared to the negative control (DMSO) was used as a cut-off for further evaluating the level of DNA damage. After electrophoresis (DNA unwinding: 20 min; electrophoresis: 20 min, 0.78 V cm^−1^, 300 mA), nucleoids stained with ethidium bromide (75 µL, 10 µg/mL) were examined with a Leica DMLS fluorescence microscope (excitation filter BP 515–560 nm, barrier filter LP 580 nm), using the software Comet Assay IV (Perceptive Instruments Ltd., Suffolk, UK). Alkaline Comet assay results are expressed as tail intensity percentage that measures the percentage of total cellular DNA found in the tail of each comet.

##### Mutagenicity Assessment on Bacteria

Mutagenicity of molecules was assessed by the standard plate incorporation method of Ames test [37] with *S. typhimurium* TA98 and TA100 strains, with and without metabolic activation (S9 mix). Compounds were dissolved in DMSO and assayed at increasing doses (0.1, 1, 10, 50 and 100 µM/plate). DMSO was used as negative control. Positive controls were 2-nitrofluorene (10 µg/plate) and sodium azide (10 µg/plate) for TA98 without S9 and TA100 without S9, respectively, and 2-aminofluorene (20 µg/plate) for both strains with S9. After 72 h, the revertant colonies grown on the plates were counted and the mean of three replicates were computed with their relative standard deviation (revertants/plate). The results were expressed as MR dividing the revertants/plate by spontaneous mutation rate (number of revertants in negative controls). The results of the test were considered positive if two consecutive dose levels or the highest non-toxic dose level produced a response at least twice that of control and at least two of these consecutive doses showed a dose-response relationship [38,39].

##### Genotoxicity Assessment on Plants

*A. cepa* toxicity test: Twelve equal-sized young onion bulbs were exposed for 96 h in the dark to different concentrations of compounds dissolved in DMSO, changing the sample solution every day. Root length was used to calculate the EC_50_ value of each compound and to identify the concentration to undergo the *A. cepa* genotoxicity test being the highest dose correspondent to the EC_50_ value identified (the concentration that gives a 50% reduction in root growth). Root macroscopic parameters such as turgescence, consistency, change in color and root tip shape, were used as toxicity indexes [40].

##### *A. cepa* Genotoxicity Tests

To detect micronuclei and chromosomal aberrations six equal-sized pre-germinated young bulbs per sample were exposed for 24 h to sample dilutions. The bulbs dedicated to the micronuclei test underwent an additional treatment in the saline solution for 44 h of recovery time to cover two rounds of mitosis so that damage induced in chromosomes during mitosis to be visible as micronuclei in interphase cells. Then all the roots were fixed in acetic acid and ethanol (1:3) for 24 h and lastly stored in 70% ethanol [41,42]. The negative control was DMSO in Rank’s solution (the dose of DMSO corresponding to volume of samples) and positive control was maleic hydrazide (10^−2^ M, 6 h exposure). Five roots of each sample were considered for microscopic analysis: 1000 cells/slide (5000 cells/sample) were scored for mitotic index (as a measure of cell division and hence of sample toxicity), 2000 in interphase cells/slide (10,000 cells/sample) were scored for micronuclei frequency and 1000 cells in mitosis (200 cells in mitosis/slide) for CA frequency were scored. All experiments were performed in duplicate.

#### 4.2.4. Statistical Analysis

Antifungal and anti-aflatoxigenic activity data were analyzed with “Past 3.x” software to determine statistically significative differences between treatments. Analysis of variance was performed by Levene test, then Kruskal–Wallis test was performed (*p* < 0.05).

Statistical analysis of cytotoxicity and genotoxicity assay on human cells was performed by the “IBM SPSS Statistics 24” software that analyses statistical differences between samples. The mean values from the repeated experiments were used in a one-way analysis of variance (ANOVA). If significant *F*-values (*p* < 0.05) were obtained, Student’s t-test (Bonferroni’s version) was performed.

Statistical analysis of mutagenicity assessment on bacteria was performed using linear regression on dose-response curve. The analysis of results of genotoxicity test on plants was performed using Chi-square test for mitotic index and chromosomal aberrations; a one-way analysis of variance (ANOVA) and Dunnett’s t-test were performed for MN analysis. All experiments were performed at least in duplicate (two independent assays).

## 5. Conclusions

To our knowledge, the AFLATOX^®^ Project is the widest screening project ever realized on the synthesis and further evaluation of antifungal and anti-aflatoxigenic properties of thiosemicarbazones obtained from naturally occurring molecules. Albeit their mechanism of action is still debated, some results ascribe the antifungal and anti-mycotoxigenic activity of TSs to the ability of modifying the cell redox balance, acting as antioxidant or ROS-stimulating agents. Other studies revealed that some TSs act at the level of lipids metabolism, influencing the regulation and biosynthesis of ergosterol and thus interfering with cell membrane formation and properties [52]. As a general consideration, it should be noted that such kind of molecules have the advantage of being much smaller and of facile high-yield syntheses if compared to AF inhibitors such as blasticidin S [53,54] and aflastatin [55,56], representing an emerging weapon in the battle against mycotoxin contamination of food and feed commodities, in field as well as during storage. Furthermore, by providing a huge amount of structured biological data, the development of the AFLATOX^®^ Project demonstrated the key role of the sulfur atom of TS in determining anti-aflatoxigenic properties. Stressing again the sulfur role, the results here reported suggested that new skeletons possessing thiophene moieties, as 2-actpTS, may provide valuable leads for the synthesis and development of novel antimicrobial agents, apparently without any cue of induced cytotoxicity and/or genotoxicity on human cell. In this sense, 2-actpTS could be considered as the best candidate for the formulation of aflatoxin control agents to be applied the field; however, in order to evaluate the suitability of the application of this compound on crops, additional in vivo tests are required—for example on insects and/or other animal models for the environment safety assessment. Another general consideration that we would like to raise at the end of the AFLATOX^®^ Project, and thus find confirmation in the data here presented about the metal complex of 2-actpTS and 2-acfurTS, is focused on the role that metal ions could play in the synthesis of new anti-aflatoxigenic compounds of practical use: even if coordination to Cu(II) and Zn(II) could enhance the anti-aflatoxigenic properties of the uncomplexed organic molecule, the metal ions tend also to introduce unacceptable cytotoxic characters. Then, further efforts should be addressed to explore other metals to be coordinated to TS, with lower toxicity on human cells and non-target organisms.

## Figures and Tables

**Table 1:** Results of the Ames test using TA98 and TA100 *S. typhimurium* strains, with and without S9, treated with 2-actpTS. Revertants/plate (mean ± S.D.) and mutagenicity ratio (MR) are reported. Negative control (NC): DMSO 100 µM/plate. Positive control (PC) –S9: TA98 10 µg/plate 2-nitrofluorene; TA100 10 µg/plate sodium azide; PC +S9: TA98, TA100 20 µg/plate 2-aminofluorene. PC results: TA 98 ± S9 > 1000 revertants/plate; TA100 ± S9 > 1000 revertants/plate.

DOSE (µM/Plate)	TA98	MR	TA98 + S9	MR
NC	15.7 ± 4.9	1.0	26.5 ± 5.4	1.0
0.1	29.5 ± 3.5	1.9	25.0 ± 0.0	0.9
1.0	23.5 ± 6.4	1.5	37.0 ± 4.2	1.4
10.0	16.0 ± 1.4	1.0	34.0 ± 2.8	1.3
50.0	22.5 ± 3.5	1.4	31.5 ± 6.4	1.2
100.0	21.0 ± 0.0	1.3	32.0 ± 2.8	1.2
	**TA100**	**MR**	**TA100 + S9**	**MR**
NC	95.8 ± 5.5	1.0	106.5 ± 11.9	1.0
0.1	92.0 ± 14.1	1.0	98.0 ± 24.0	0.9
1.0	101.5 ± 4.9	1.1	104.0 ± 15.6	1.0
10.0	104.5 ± 9.2	1.1	107.5 ± 0.7	1.0
50.0	87.5 ± 7.8	0.9	90.5 ± 13.4	0.9
100.0	99.5 ± 0.7	1.0	88.0 ± 8.5	0.8

**Table 2:** Roots length, micronuclei and chromosomal aberrations frequency in *A. cepa* treated with 2-actpTS. Root length is expressed as cm (mean ± S.D.); micronuclei (MN) frequency is expressed as % (mean ± S.D.) and chromosomal aberrations (CA) are expressed as the ratio of number of aberrated cells to the total cells in mitosis (%). Positive control: 10^−2^ M maleic hydrazide (MN 16.4 ± 5.9; CA 8.8).

	Toxicity Test	Genotoxicity Test	
Doses (µM)	Roots Length	MN	CA
NC	2.07 ± 0.61	1.10 ± 1.28	1.2
10	2.17 ± 0.53	1.20 ± 0.83	2.7
25	1.65 ± 0.43	1.40 ± 1.14	4.7
50	1.68 ± 0.39	2.00 ± 1.58	6.3
100	1.07 ± 0.23	-	6.0

## Data Availability

The database presented in this study are openly available at https://aflatox.unibs.it (accessed on 26 April 2021).

## Supplementary Materials

The following are available online at https://www.mdpi.com/article/10.3390/ijms22094520/s1, Table S1: Compounds effective in reducing up to 50% the growth of *A. flavus*, when administrated at 50 and 100 µM, Table S2: Compounds effective in reducing up to 50% the aflatoxin accumulation, when administrated at 50 and 100 µM, Table S3: Growth inhibition data obtained on Crl1790, Hs27, HFL1 and U937 cells after 24 h treatment with different concentration (0–100 µM) of 2-actpTS. Data are expressed as mean ± standard deviation of three independent experiments, Table S4: Growth inhibition data obtained on Hs27 cells after 24 h treatment with different concentration (0–100 µM) of 2-acfurTS-Cu, 2-acfurTS-Zn, 2-actpTS-Cu and 2-actpTS-Zn. Data are expressed as mean ± standard deviation of three independent experiments.
