# Supplementary material for: The AFLATOX® Project: Approaching the Development of New Generation, Natural-Based Compounds for the Containment of the Mycotoxigenic Phytopathogen Aspergillus flavus and Aflatoxin Contamination
Source: Int J Mol Sci. 2021 Apr 26;22(9):4520. doi: 10.3390/ijms22094520 (PMC8123576; doi:10.3390/ijms22094520)
Supplement: Supplementary file 1 [file ijms-22-04520-s001.zip › Table 1S.pdf]

Compounds effective in inhibiting the *A. flavus* growth more than 50% at the concentration 50 µM					
**Compound**	**Structure**	**MW**	**Formula**	**Group**	**G.I. (%)**
Tosylate -2-methyleneanthraquinone	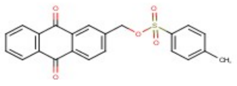	392,43	C22H16O5S	Anthraquinone	66,0
3-Phenylpropanal thiosemicarbazone	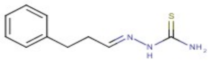	207,30	C10H13N3S	Cinnamaldehyde	100
Cu(II) cinnamaldehyde bisthiosemicarbazonate	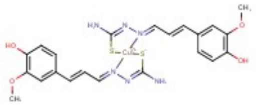	564,14	C22H24CuN6O4S2	Cinnamaldehyde	58,0
Cu(II) 3-phenylpropanal bisthiosemicarbazonate	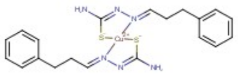	476,12	C20H24CuN6S2	Cinnamaldehyde	69,0
Ni(II) Cinnamaldehyde bisthiosemicarbazonate	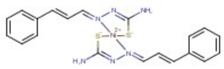	467,24	C20H20N6NiS2	Cinnamaldehyde	87,9
Zn(II) Cinnamaldehyde bisthiosemicarbazonate	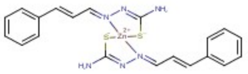	473,92	C20H20N6S2Zn	Cinnamaldehyde	56,0
(E)-1-(4-methoxyphenyl)-1-pentene-3-one-3-thiosemicarbazone	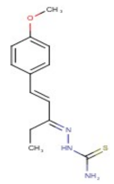	263,36	C13H17N3OS	Cinnamaldehyde	62,0
Zn(II) (E)-1-(4-methoxyphenyl)-1-pentene-3-one-bis 3-thiosemicarbazonate	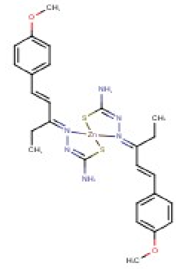	590,08	C26H32N6O2S2Zn	Cinnamaldehyde	86,0
Cuminaldehyde thiosemicarbazone	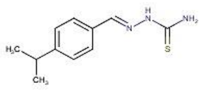	221,32	C11H15N3S	Cuminaldehyde	80,0
Ni(II) Cuminaldehyde bisthiosemicarbazonate	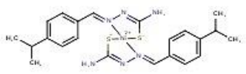	499,32	C22H28N6NiS2	Cuminaldehyde	84,0

Zn(II) Cuminaldehyde bisthiosemicarbazonate	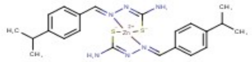	506,01	$C_{22}H_{28}N_6S_2Zn$	Cuminaldehyde	56,0
Cu(II) bis acetate 2-isopropylbenzaldehyde bisthiosemicarbazone	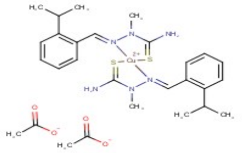	652,33	$C_{28}H_{40}CuN_6O_4S_2$	Cuminaldehyde	90,0
Cyclohexanone thiosemicarbazone	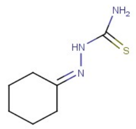	171,26	$C_7H_{13}N_3S$	Cyclohexanone	80,0
Dicyclohexanone thiosemicarbazone	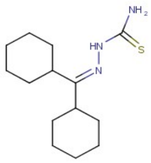	267,44	$C_{14}H_{25}N_3S$	Dicyclohexyl ketone	90,0
Decanal thiosemicarbazone	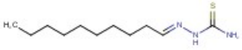	229,39	$C_{11}H_{23}N_3S$	Decanal	96,0
Cu(II) 2,4-decadienal bisthiosemicarbazonate	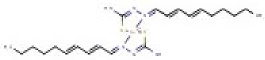	512,24	$C_{22}H_{36}CuN_6S_2$	Decanal	91,0
Flavone	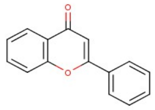	222,24	$C_{15}H_{10}O_2$	Flavone	66,0
Flavone thiosemicarbazone	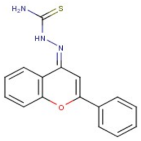	295,36	$C_{16}H_{13}N_3OS$	Flavone	100
Cu(II) 4,7'-dihydroxyflavone bisthiosemicarbazonate	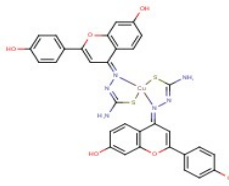	716,25	$C_{32}H_{24}CuN_6O_6S_2$	Flavone	100
Cu(I) chloroaqua flavone thiosemicarbazone	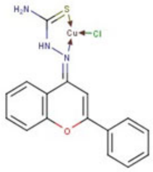	395,36	$C_{16}H_{14}ClCuN_3OS$	Flavone	60,0

Nonanal thiosemicarbazone	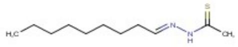	214,37	C11H22N2S	Nonanal	95,0
2-Octenal thiosemicarbazone	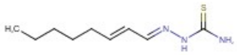	199,32	C9H17N3S	Octanal	93,0
Octanal thiosemicarbazone	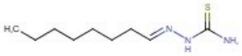	201,33	C9H19N3S	Octanal	82,0
2,3-dihydroxybenzaldehyde thiosemicarbazone	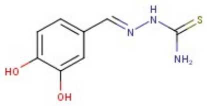	211,24	C8H9N3O2S	Protocatechol-aldehyde	53,0
Cu(II) 2,3,4-trimethoxybenzaldehyde bisthiosemicarbazonate	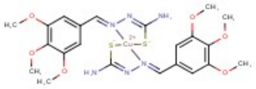	600,17	C22H28CuN6O6S2	Syringaldehyde	55,0
Undecanal thiosemicarbazone	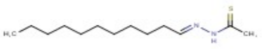	242,43	C13H26N2S	Undecanal	78,0
Cu(II)undecanal bisthiosemicarbazonate	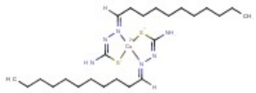	548,36	C24H48CuN6S2	Undecanal	60,4
Cu(II) apocynin bisthiosemicarbazonate	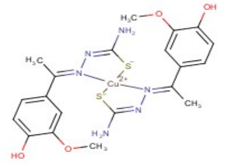	540,12	C20H24CuN6O4S2	Vanillin	61,0

Compounds effective in inhibiting the *A. flavus* growth more than 50% at the concentration 100 µM

Compound	Structure	MW	Formula	Group	G.I. (%)
Acetylthiophene bisthiosemicarbazone	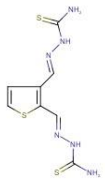	286,39	C8H10N6S3	2-Acetylthiophene	65,0
Cu(II) 2-acetylfurane bisthiosemicarbazonate	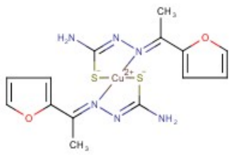	427,99	C14H16CuN6O2S2	2-Acetylthiophene	63,4

Cu(II) acetylthiophene bisthiosemicarbazonate	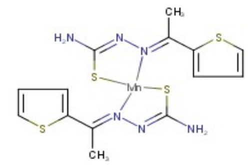	451,50	C14H16MnN6S4	2-Acetylthiophene	53,0
2-hydroxymethyl-anthraquinone	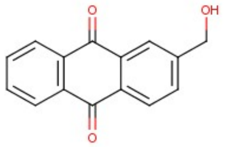	238,24	C15H10O3	Anthraquinone	62,0
2-Formylanthraquinone	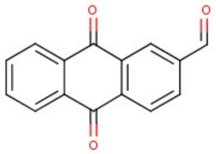	236,23	C15H8O3	Anthraquinone	58,0
Anthraquinone-2-carbonyl-1-thiosemicarbazide	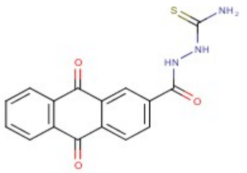	325,34	C16H11N3O3S	Anthraquinone	58,0
Tosylate -2-methyleneanthraquinone	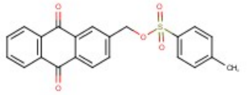	392,43	C22H16O5S	Anthraquinone	87,0
Benzophenone thiosemicarbazone	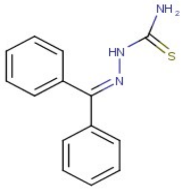	255,34	C14H13N3S	Benzophenone	53,0
Camphorquinone bis thiosemicarbazone	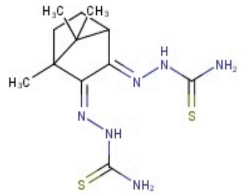	312,45	C12H20N6S2	Camphorquinone	58,0
3-Phenylpropanal thiosemicarbazone	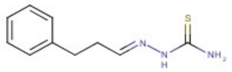	207,30	C10H13N3S	Cinnamaldehyde	107,0
Cu(II) 3-phenylpropanal bisthiosemicarbazonate	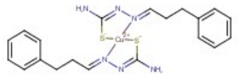	476,12	C20H24CuN6S2	Cinnamaldehyde	68,0
Cinnamaldehyde thiosemicarbazone	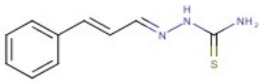	205,28	C10H11N3S	Cinnamaldehyde	56,0

Ni(II) Cinnamaldehyde bisthiosemicarbazonate	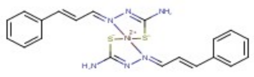	467,24	C20H20N6NiS2	Cinnamaldehyde	100
Zn(II) Cinnamaldehyde bisthiosemicarbazonate	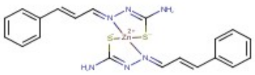	473,92	C20H20N6S2Zn	Cinnamaldehyde	76,0
(E)-1-(4-methoxyphenyl)-1-pentene-3-one-3-thiosemicarbazone	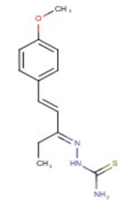	263,36	C13H17N3OS	Cinnamaldehyde	74,0
Zn(II) (E)-1-(4-methoxyphenyl)-1-pentene-3-one-bis 3-thiosemicarbazonate	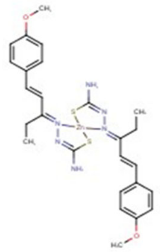	590,08	C26H32N6O2S2Zn	Cinnamaldehyde	89,0
Citral thiosemicarbazone	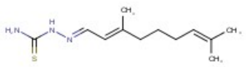	239,38	C12H21N3S	Citral	57,0
Ni(II) citronellal bisthiosemicarbazonate	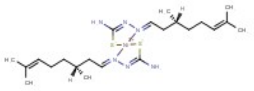	511,42	C22H40N6NiS2	Citronellal	58,9
Cyclohexanone thiosemicarbazone	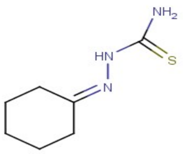	171,26	C7H13N3S	Cyclohexanone	95,0
Cuminaldehyde thiosemicarbazone	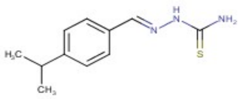	221,32	C11H15N3S	Cuminaldehyde	90,0
Ni(II) Cuminaldehyde bisthiosemicarbazonate	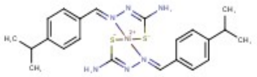	499,32	C22H28N6NiS2	Cuminaldehyde	90,0
Zn(II) Cuminaldehyde bisthiosemicarbazonate	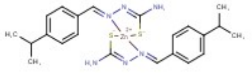	506,01	C22H28N6S2Zn	Cuminaldehyde	60,3

3-isopropyl benzaldehyde-2-methyl-thiosemicarbazone	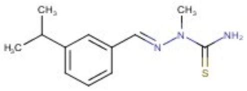	235,35	C12H17N3S	Cuminaldehyde	70,6
Cu(II) 2-isopropyl benzaldehyde-2-methyl-thiosemicarbazone di acetate	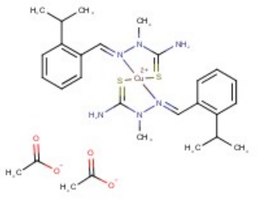	652,33	C28H40CuN6O4S2	Cuminaldehyde	95
Decanal thiosemicarbazone	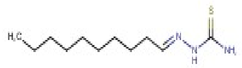	229,39	C11H23N3S	Decanal	96,0
Cu(II) 2,4-decadienal bisthiosemicarbazonate	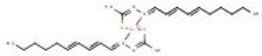	512,24	C22H36CuN6S2	Decanal	80,0
Dicyclohexanone thiosemicarbazone	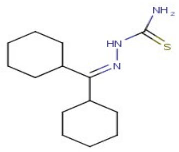	267,44	C14H25N3S	Dicyclohexyl ketone	99,00
Cu(II) fenchone bisthiosemicarbazonate	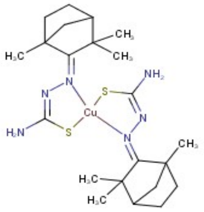	512,24	C22H36CuN6S2	Fenchone	57,0
Flavone	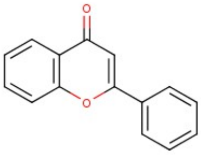	222,24	C15H10O2	Flavone	83,0
Flavone thiosemicarbazone	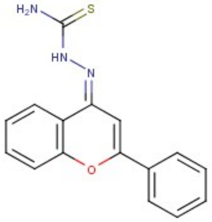	295,36	C16H13N3OS	Flavone	100
Cu(II) 4,7'-dihydroxyflavone bisthiosemicarbazonate	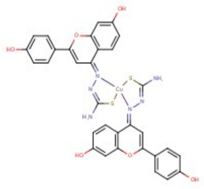	716,25	C32H24CuN6O6S2	Flavone	100

Cu(I) chloro aqua flavonethiosemicarbazone	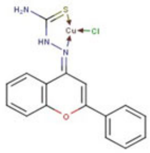	395,36	C16H14ClCuN3OS	Flavone	79,0
Heptanal thiosemicarbazone	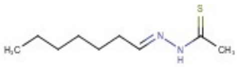	186,32	C9H18N2S	Heptanal	58,00
Cu(II) jasmone bisthiosemicarbazonate	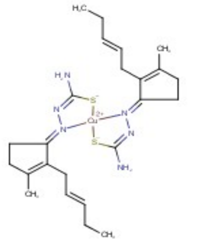	536,26	C24H36CuN6S2	Jasmone	74,3
Nonanal thiosemicarbazone	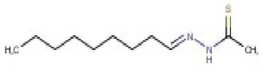	214,37	C11H22N2S	Nonanal	100
Cu(II) nonanal bisthiosemicarbazonate	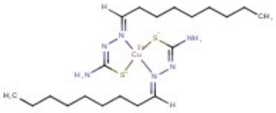	492,25	C20H40CuN6S2	Nonanal	84,1
2-Octenal thiosemicarbazone	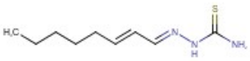	199,32	C9H17N3S	Octanal	96,0
Octanal thiosemicarbazone	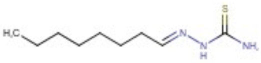	201,33	C9H19N3S	Octanal	93,0
Perillaldehyde thiosemicarbazone	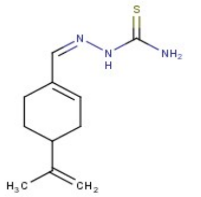	223,34	C11H17N3S	Perillaldehyde	73,5
Plumbagin thiosemicarbazone	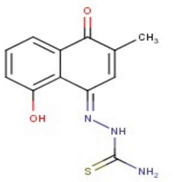	261,30	C12H11N3O2S	Plumbagin	61,9
3,4-dihydroxybenzaldehyde thiosemicarbazone	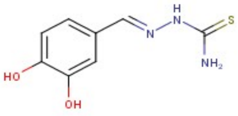	211,24	C8H9N3O2S	Protocatechol-aldehyde	77,0

Cu(II) Quinolinecarboxaldehyde bisthiosemicarbazonate	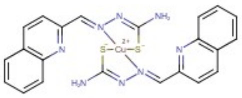	522,11	C22H18CuN8S2	2-Quinoline carboxaldehyde	60,7
Cu(II) syringaldehyde bisthiosemicarbazonate	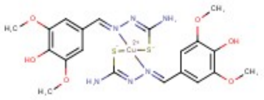	572,11	C20H24CuN6O6S2	Syringaldehyde	61,0
Cu(II) 3,4,5-trimethoxybenzaldehyde bisthiosemicarbazonate	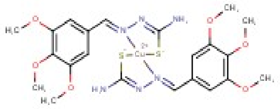	600,17	C22H28CuN6O6S2	Syringaldehyde	54,0
Cu(II) undecanal bisthiosemicarbazonate	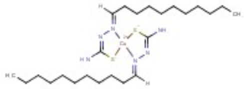	548,36	C24H48CuN6S2	Undecanal	94,0
Undecanal thiosemicarbazone	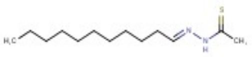	242,43	C13H26N2S	Undecanal	62,0
Valerophenone thiosemicarbazone	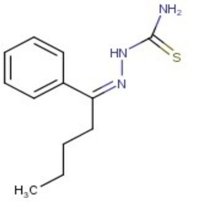	235,35	C12H17N3S	Valerophenone	54,0
Cu(II) apocynin bisthiosemicarbazonate	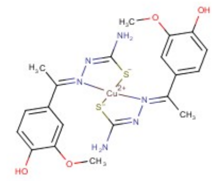	540,12	C20H24CuN6O4S2	Vanillin	72,0
Cu(II) 3,4-dimethoxybenzaldehyde bisthiosemicarbazonate	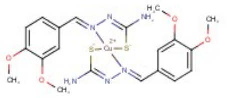	540,12	C20H24CuN6O4S2	Vanillin	51,0
Bisvanillin thiosemicarbazone	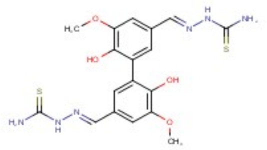	448,52	C18H20N6O4S2	Vanillin	57,0
Cu(II) bisvanillin thiosemicarbazonate	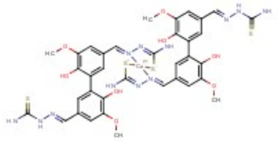	958,56	C36H38CuN12O8S4	Vanillin	65,5
