# Supplementary material for: The AFLATOX® Project: Approaching the Development of New Generation, Natural-Based Compounds for the Containment of the Mycotoxigenic Phytopathogen Aspergillus flavus and Aflatoxin Contamination
Source: Int J Mol Sci. 2021 Apr 26;22(9):4520. doi: 10.3390/ijms22094520 (PMC8123576; doi:10.3390/ijms22094520)
Supplement: Supplementary file 1 [file ijms-22-04520-s001.zip › Table 2S.pdf]

Table 2S

Compounds effective in inhibiting aflatoxin accumulation more than 50% at the concentration 50 μM					
**Compound**	**Structure**	**MW**	**Formula**	**Group**	**A.I. (%)**
2-acetylthiophene thiosemicarbazone	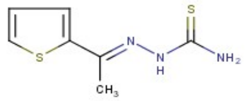	199,29	C7H9N3S2	2-Acetylthiophene	58,3
Cu(II) 2-acetylthiophene bisthiosemicarbazonate	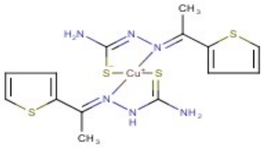	461,12	C14H17CuN6S4	2-Acetylthiophene	56,5
Zn(II) 2-acetylthiophene bisthiosemicarbazonate	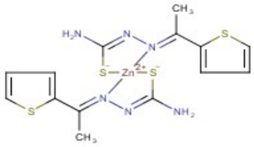	461,94	C14H16N6S4Zn	2-Acetylthiophene	60,4
Mn(II) 2-acetylthiophene bisthiosemicarbazonate	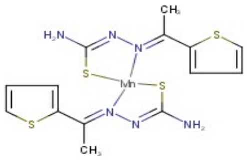	451,50	C14H16MnN6S4	2-Acetylthiophene	61,0
Cu(II) Quinoline carboxaldehyde bisthiosemicarbazonate	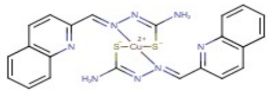	522,11	C22H18CuN8S2	2-Quinolinecarbox-aldehyde	55,7
Anthraquinone-2-carboxylic acid	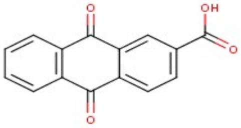	252,23	C15H8O4	Anthraquinone	56,0
benzaldehyde 4,4'-dimethyl thiosemicarbazone	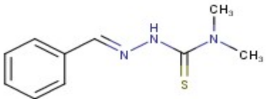	207,30	C10H13N3S	Benzaldehyde	76,0
Cu(II) 1-(3-methoxyphenyl)heptan-1-one bisthiosemicarbazonate	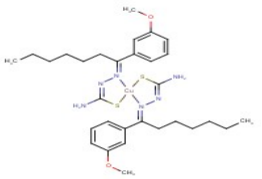	648,39	C30H44CuN6O2S2	Benzaldehyde	82,5

Cinnamaldehyde thiosemicarbazone	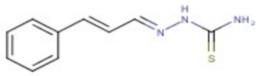	205,28	$C_{10}H_{11}N_3S$	Cinnamaldehyde	73,1
Ni(II) Cinnamaldehyde bisthiosemicarbazonate	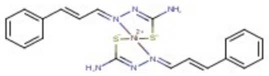	467,24	$C_{20}H_{20}N_6NiS_2$	Cinnamaldehyde	95,0
Zn(II) Cinnamaldehyde bisthiosemicarbazonate	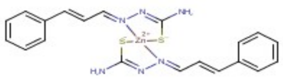	473,92	$C_{20}H_{20}N_6S_2Zn$	Cinnamaldehyde	99,0
Cinnamaldehyde 4,4-dimethyl thiosemicarbazone	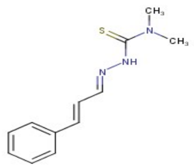	233,33	$C_{12}H_{15}N_3S$	Cinnamaldehyde	100
Zn(II) Cinnamaldehyde bis 4,4-dimethyl thiosemicarbazonate	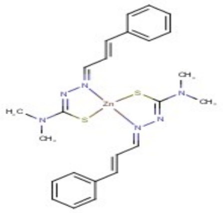	530,03	$C_{24}H_{28}N_6S_2Zn$	Cinnamaldehyde	75,0
Zn(II) 1-isopropylbenzaldehyde bisthiosemicarbazonate	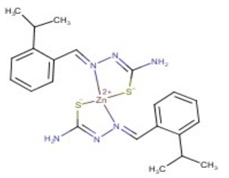	506,01	$C_{22}H_{28}N_6S_2Zn$	Cuminaldehyde	55,3
Cuminaldehyde thiosemicarbazone	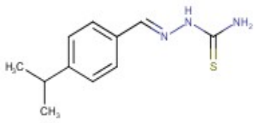	221,32	$C_{11}H_{15}N_3S$	Cuminaldehyde	100
Ni(II) Cuminaldehyde bisthiosemicarbazonate	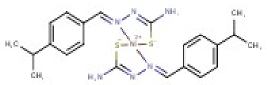	499,32	$C_{22}H_{28}N_6NiS_2$	Cuminaldehyde	91,5
Zn(II) 2-isopropylbenzaldehyde bisthiosemicarbazonate	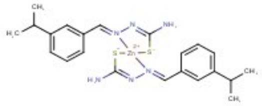	506,01	$C_{22}H_{28}N_6S_2Zn$	Cuminaldehyde	93,8

Zn(II) 1-isopropylbenzaldehyde bis thiosemicarbazone diacetate	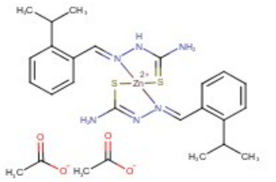	625,10	C26H35N6O4S2Zn	Cuminaldehyde	69,3
2-isopropylbenzaldehyde thiosemicarbazone	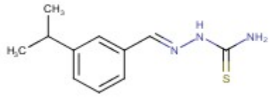	221,32	C11H15N3S	Cuminaldehyde	67,0
Cu(II) 2-isopropylbenzaldehyde bisthiosemicarbazonate	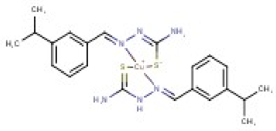	505,18	C22H29CuN6S2	Cuminaldehyde	65,9
Zn(II) Cuminaldehyde bisthiosemicarbazonate	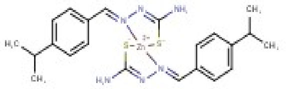	506,01	C22H28N6S2Zn	Cuminaldehyde	97,1
Cu(II) 2-isopropyl benzaldehyde-2-methyl-thiosemicarbazone di acetate	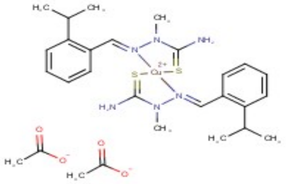	652,33	C28H40CuN6O4S2	Cuminaldehyde	71,4
Cu(II) fenchone bisthiosemicarbazonate	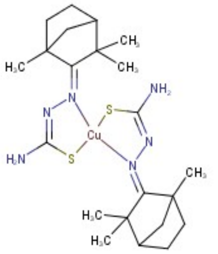	512,24	C22H36CuN6S2	Fenchone	62,0
Cu(II) heptanal bisthiosemicarbazonate	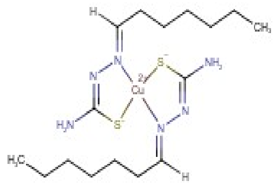	436,14	C16H32CuN6S2	Heptanal	62,8
Dihydrojasmone thiosemicarbazone	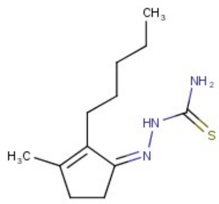	239,38	C12H21N3S	Jasmone	51,5

Jasmone thiosemicarbazone	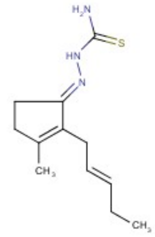	237,37	C12H19N3S	Jasmone	88,0
Cu(II) Dihydrojasmone bisthiosemicarbazonate	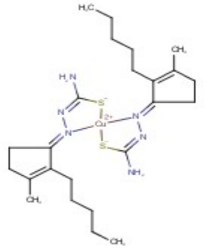	540,29	C24H40CuN6S2	Jasmone	66,0
Cu(II) jasmone bisthiosemicarbazonate	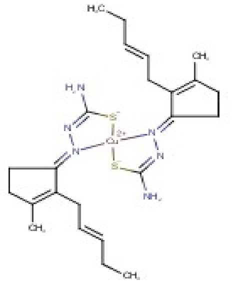	536,26	C24H36CuN6S2	Jasmone	78,0
Cu(II) nonanal bisthiosemicarbazonate	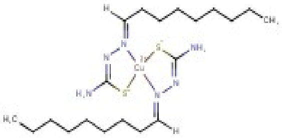	492,25	C20H40CuN6S2	Nonanal	90,0
Nonanal thiosemicarbazone	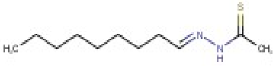	214,37	C11H22N2S	Nonanal	91,0
Octanal thiosemicarbazone	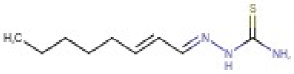	199,32	C9H17N3S	Octanal	76,0
Perillaldehyde thiosemicarbazone	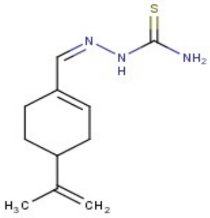	223,34	C11H17N3S	Perillaldehyde	78,0

Ni(II) perillaldehyde bisthiosemicarbazonate	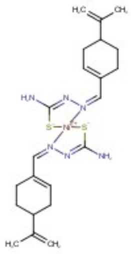	503,35	C22H32N6NiS2	Perillaldehyde	85,2
Cu(II) undecanal bisthiosemicarbazonate	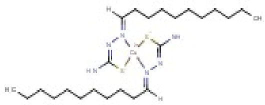	548,36	C24H48CuN6S2	Undecanal	84,8
Undecanal thiosemicarbazone	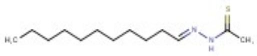	242,43	C13H26N2S	Undecanal	72,0
Cu(II) apocynin bisthiosemicarbazonate	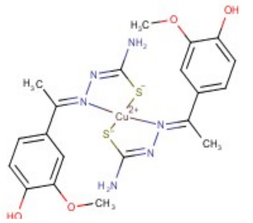	540,12	C20H24CuN6O4S2	Vanillin	52,0
Cu(II) 3,4-dimethoxybenzaldehyde bisthiosemicarbazonate	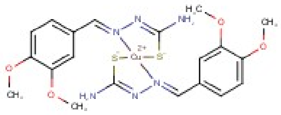	540,12	C20H24CuN6O4S2	Vanillin	62,0
Cu(II) bisvanillin thiosemicarbazonate	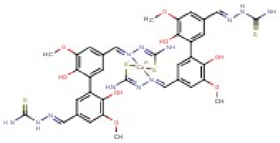	958,56	C36H38CuN12O8S4	Vanillin	53,0
Cu(II) 3,4-dimethoxybenzaldehyde bis 4,4'-dimethyl thiosemicarbazonate	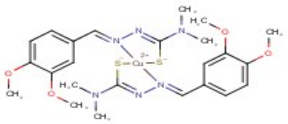	596,22	C24H32CuN6O4S2	Vanillin	61,4

Compounds effective in inhibiting aflatoxin accumulation more than 50% at the concentration 100 μM

Compound	Structure	MW	Formula	Group	A.I. (%)
2-acetylthiophene thiosemicarbazone	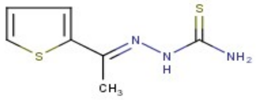	199,29	C7H9N3S2	2-Acetylthiophene	88,4

Cu(II) 2-acetylfurane bisthiosemicarbazonate	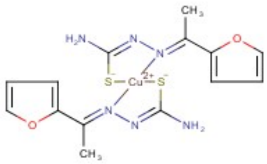	427,99	C14H16CuN6O2S2	2-Acetylthiophene	65,7
Cu(II) 2-acetylthiophene bisthiosemicarbazonate	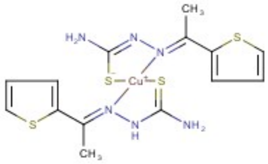	461,12	C14H17CuN6S4	2-Acetylthiophene	88,6
Zn(II) 2-acetylthiophene bisthiosemicarbazonate	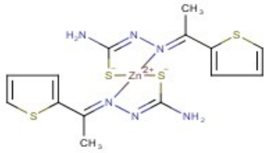	461,94	C14H16N6S4Zn	2-Acetylthiophene	85,4
Cu(II) acetylthiophene bisthiosemicarbazonate	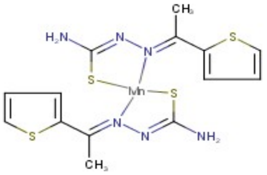	451,50	C14H16MnN6S4	2-Acetylthiophene	92,0
Cu(II) Quinoline carboxaldehyde bisthiosemicarbazonate	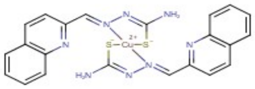	522,11	C22H18CuN8S2	2-Quinoline carboxaldehyde	88,0
Anthraquinone-2-carboxylic acid	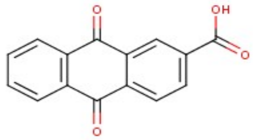	252,23	C15H8O4	Anthraquinone	80,0
Benzaldehyde 4,4'-dimethyl thiosemicarbazone	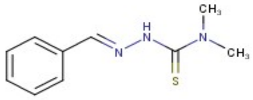	207,30	C10H13N3S	Benzaldehyde	90,0
Cu(II) benzaldehyde 4,4'-dimethyl thiosemicarbazonate	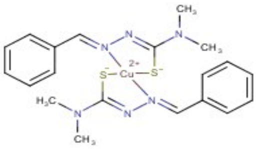	476,12	C20H24CuN6S2	Benzaldehyde	67,0
Cu(II) 1-(3-methoxyphenyl) heptan-1-one bisthiosemicarbazonate	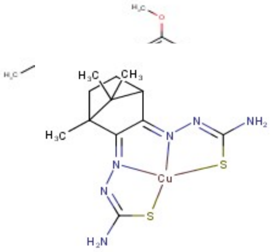	648,39	C30H44CuN6O2S2	Benzaldehyde	95,2

Cu(II) Camphorquinone bisthiosemicarbazonate		373,98	C12H18CuN6S2	Camphorquinone	53,0
Cinnamaldehyde thiosemicarbazone	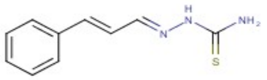	205,28	C10H11N3S	Cinnamaldehyde	79,0
Ni(II) Cinnamaldehyde bisthiosemicarbazonate	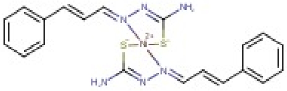	467,24	C20H20N6NiS2	Cinnamaldehyde	100
Zn(II) Cinnamaldehyde bisthiosemicarbazonate	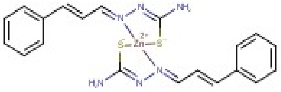	473,92	C20H20N6S2Zn	cinnamaldehyde	100
Cinnamaldehyde 4,4-dimethyl thiosemicarbazone		233,33	C12H15N3S	cinnamaldehyde	100
Zn(II) Cinnamaldehyde bis 4,4-dimethyl thiosemicarbazonate	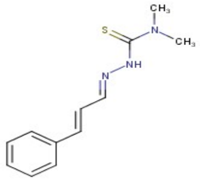 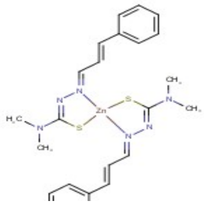	530,03	C24H28N6S2Zn	Cinnamaldehyde	100
Cinnamaldehyde 2-methyl thiosemicarbazone	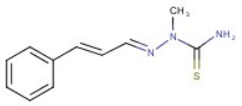	219,31	C11H13N3S	Cinnamaldehyde	64,0
Ni(II) citronellal bisthiosemicarbazonate	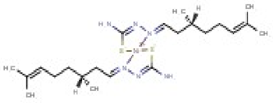	511,42	C22H40N6NiS2	Citronellal	82,1

Zn(II) 1-isopropylbenzaldehyde bisthiosemicarbazonate	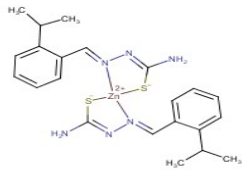	506,01	C22H28N6S2Zn	Cuminaldehyde	100
Cuminaldehyde thiosemicarbazone	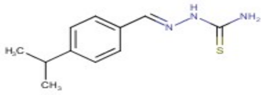	221,32	C11H15N3S	Cuminaldehyde	100
Ni(II) Cuminaldehyde bisthiosemicarbazonate	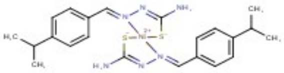	499,32	C22H28N6NiS2	Cuminaldehyde	96,0
Zn(II) 2-isopropylbenzaldehyde bisthiosemicarbazonate	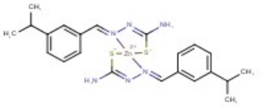	506,01	C22H28N6S2Zn	Cuminaldehyde	100
Zn(II) 1-isopropylbenzaldehyde bis thiosemicarbazone diacetate	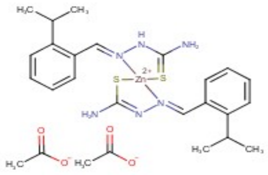	625,10	C26H35N6O4S2Zn	Cuminaldehyde	81,9
2-isopropylbenzaldehyde thiosemicarbazone	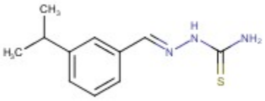	221,32	C11H15N3S	Cuminaldehyde	97,0
Cu(II) 1-isopropylbenzaldehyde bisthiosemicarbazonate	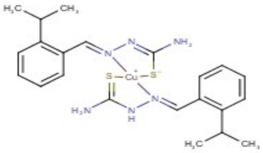	505,18	C22H29CuN6S2	Cuminaldehyde	73,5
Cu(II) 2-isopropylbenzaldehyde bisthiosemicarbazonate	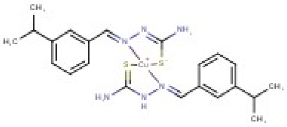	505,18	C22H29CuN6S2	Cuminaldehyde	80,8
Zn(II) Cuminaldehyde bisthiosemicarbazonate	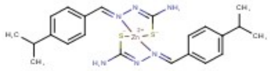	506,01	C22H28N6S2Zn	Cuminaldehyde	100

3-isopropyl benzaldehyde-2-methyl-thiosemicarbazone	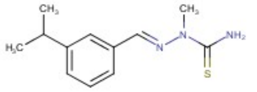	235,35	C12H17N3S	Cuminaldehyde	62,7
Cu(II) 2-isopropyl benzaldehyde-2-methyl-thiosemicarbazone di acetate	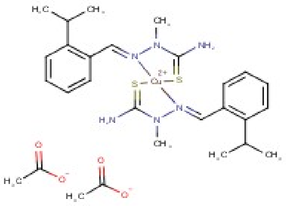	652,33	C28H40CuN6O4S2	Cuminaldehyde	86,7
Decanal thiosemicarbazone	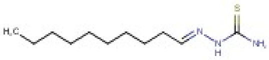	229,39	C11H23N3S	Decanal	62,0
Cu(II) fenchone bisthiosemicarbazonate	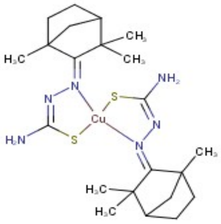	512,24	C22H36CuN6S2	Fenchone	84,0
Cu(II) heptanal bisthiosemicarbazonate	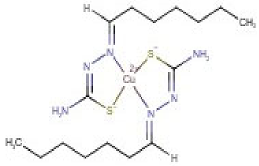	436,14	C16H32CuN6S2	Heptanal	85,5
Heptanal thiosemicarbazone	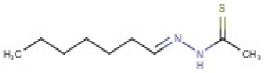	186,32	C9H18N2S	Heptanal	51,0
Valerophenone thiosemicarbazone	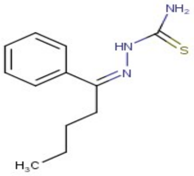	235,35	C12H17N3S	Valerophenone	55,0
Dihydrojasmone thiosemicarbazone	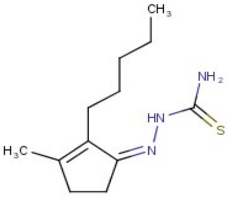	239,38	C12H21N3S	Jasmone	63,2

Jasmone thiosemicarbazone	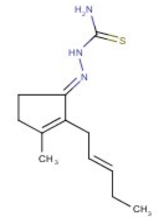	237,37	C12H19N3S	Jasmone	90,0
Cu(II) Dihydrojasmone bisthiosemicarbazonate	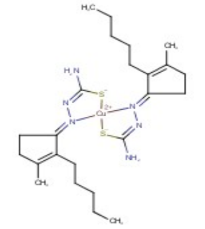	540,29	C24H40CuN6S2	Jasmone	80,0
Cu(II) jasmone bisthiosemicarbazonate	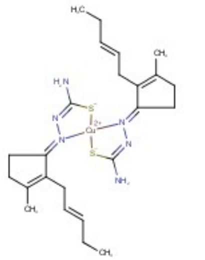	536,26	C24H36CuN6S2	Jasmone	97,0
Cu(II) nonanal bisthiosemicarbazonate	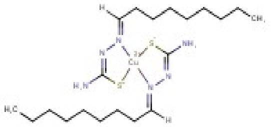	492,25	C20H40CuN6S2	Nonanal	100
Nonanal thiosemicarbazone	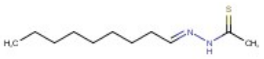	214,37	C11H22N2S	Nonanal	100
Octanal thiosemicarbazone	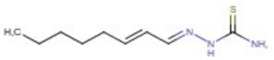	199,32	C9H17N3S	Octanal	96,0
Perillaldehyde thiosemicarbazone	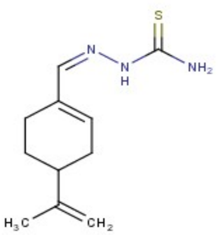	223,34	C11H17N3S	Perillaldehyde	87,3

Ni(II) Perillaldehyde bisthiosemicarbazonate	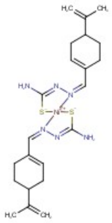	503,35	$C_{22}H_{32}N_6NiS_2$	Perillaldehyde	90,1
Plumbagin thiosemicarbazone	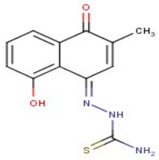	261,30	$C_{12}H_{11}N_3O_2S$	Plumbagin	83,0
Cu(II) syringaldehyde bisthiosemicarbazonate	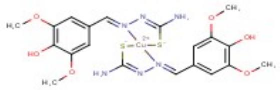	572,11	$C_{20}H_{24}CuN_6O_6S_2$	Syringaldehyde	61,0
Cu(II) 3,4,5-trimethoxybenzaldehyde bisthiosemicarbazonate	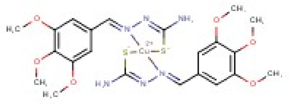	600,17	$C_{22}H_{28}CuN_6O_6S_2$	Syringaldehyde	76,0
Cu(II)undecanal bisthiosemicarbazonate	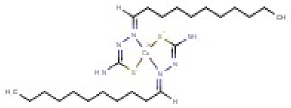	548,36	$C_{24}H_{48}CuN_6S_2$	Undecanal	96,0
Undecanal thiosemicarbazone	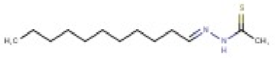	242,43	$C_{13}H_{26}N_2S$	Undecanal	93,0
Cu(II) apocynin bisthiosemicarbazonate	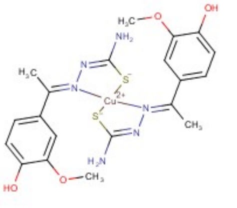	540,12	$C_{20}H_{24}CuN_6O_4S_2$	Vanillin	72,0
Cu(II) 3,4-dimethoxybenzaldehyde bisthiosemicarbazonate	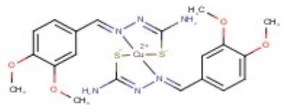	540,12	$C_{20}H_{24}CuN_6O_4S_2$	Vanillin	80,0

Bisvanillin thiosemicarbazone	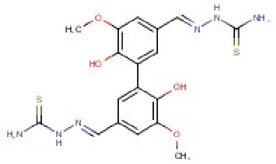	448,52	C18H20N6O4S2	Vanillin	61,0
Cu(II) bisvanillin thiosemicarbazonate	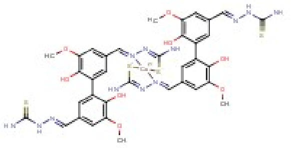	958,56	C36H38CuN12O8S4	Vanillin	77,0
Cu(II) 3,4-dimethoxybenzaldehyde bis 4,4'-dimethyl thiosemicarbazonate	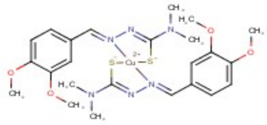	596,22	C24H32CuN6O4S2	Vanillin	84,6
