# Supplementary material for: The AFLATOX® Project: Approaching the Development of New Generation, Natural-Based Compounds for the Containment of the Mycotoxigenic Phytopathogen Aspergillus flavus and Aflatoxin Contamination
Source: Int J Mol Sci. 2021 Apr 26;22(9):4520. doi: 10.3390/ijms22094520 (PMC8123576; doi:10.3390/ijms22094520)
Supplement: Supplementary file 1 [file ijms-22-04520-s001.zip › Table 3S-4S.pdf]

Table 3S. Growth inhibition data obtained on Crl1790, Hs27, HFL1 and U937 cells after 24 h treatment with different concentration (0-100 μM) of 2-actpTS. Data are expressed as mean ± standard deviation of three independent experiments.

**Dose (μM)**	**Crl1790**	**Hs27**	**HFL1**	**U937**
**0**	100.00	100.00	100.00	100.00
**0.5**	100.00 ± 3.63	99.92 ± 7.65	100.00 ± 11.14	100.00 ± 9.43
**1**	95.09 ± 7.76	96.96 ± 4.78	100.00 ± 6.11	100.00 ± 4.23
**5**	88.36 ± 2.25	100.00 ± 5.97	100.00 ± 5.74	100.00 ± 6.71
**10**	89.11 ± 5.24	97.71 ± 3.73	100.00 ± 6.76	100.00 ± 7.06
**50**	81.76 ± 3.48	100.00 ± 9.25	100.00 ± 4.53	100.00 ± 3.60
**100**	62.04 ± 4.60	87.26 ± 8.07	92.52 ± 3.49	94.72 ± 8.15

Table 4S. Growth inhibition data obtained on Hs27 cells after 24 h treatment with different concentration (0-100 μM) of 2-acfurTS-Cu, 2-acfurTS-Zn, 2-actpTS-Cu and 2-actpTS-Zn. Data are expressed as mean ± standard deviation of three independent experiments.

**Dose (μM)**	**2-acfurTS-Cu**	**2-acfurTS-Zn**	**2-actpTS-Cu**	**2-actpTS-Zn**
**0**	100.00	100.00	100.00	100.00
**0.5**	92.82 ± 5.53	100.00 ± 7.72	90.69 ± 4.69	91.63 ± 7.33
**1**	99.42 ± 4.63	100.00 ± 7.88	87.09 ± 3.53	94.34 ± 2.25
**5**	98.87 ± 12.07	100.00 ± 7.70	72.13 ± 3.35	81.89 ± 5.34
**10**	100.00 ± 5.93	100.00 ± 12.73	16.23 ± 0.84	60.15 ± 1.57
**50**	36.86 ± 6.49	16.50 ± 0.62	14.64 ± 0.85	15.54 ± 0.56
**100**	35.65 ± 1.89	15.17 ± 0.73	15.03 ± 0.20	15.96 ± 1.39
